# Strain-specific alterations in gut microbiome and host immune responses elicited by tolerogenic *Bifidobacterium pseudolongum*

**DOI:** 10.1038/s41598-023-27706-0

**Published:** 2023-01-19

**Authors:** Bing Ma, Samuel J. Gavzy, Vikas Saxena, Yang Song, Wenji Piao, Hnin Wai Lwin, Ram Lakhan, Jegan Iyyathurai, Lushen Li, Michael France, Christina Paluskievicz, Marina W. Shirkey, Lauren Hittle, Arshi Munawwar, Emmanuel F. Mongodin, Jonathan S. Bromberg

**Affiliations:** 1 Institute of Genome Sciences, University of Maryland School of Medicine, Baltimore, MD 21201 USA; 2 Department of Microbiology and Immunology, University of Maryland School of Medicine, Baltimore, MD 21201 USA; 3 Department of Surgery, University of Maryland Medical Center, Baltimore, MD 21201 USA; 4 Center for Vascular and Inflammatory Diseases, University of Maryland School of Medicine, Baltimore, MD 21201 USA; 5 Institute of Human Virology, University of Maryland School of Medicine, Baltimore, MD 21201 USA; 6 Present Address: Division of Lung Diseases, National Heart, Lung, and Blood Institute (NHLBI), National Institutes of Health (NIH), Bethesda, MD USA

**Keywords:** Immunology, Transplant immunology, Microbiology, Microbial communities, Microbial ecology, Microbiome

## Abstract

The beneficial effects attributed to *Bifidobacterium* are largely attributed to their immunomodulatory capabilities, which are likely to be species- and even strain-specific. However, their strain-specificity in direct and indirect immune modulation remain largely uncharacterized. We have shown that *B. pseudolongum* UMB-MBP-01, a murine isolate strain, is capable of suppressing inflammation and reducing fibrosis in vivo. To ascertain the mechanism driving this activity and to determine if it is specific to UMB-MBP-01, we compared it to a porcine tropic strain *B. pseudolongum* ATCC25526 using a combination of cell culture and in vivo experimentation and comparative genomics approaches. Despite many shared features, we demonstrate that these two strains possess distinct genetic repertoires in carbohydrate assimilation, differential activation signatures and cytokine responses signatures in innate immune cells, and differential effects on lymph node morphology with unique local and systemic leukocyte distribution. Importantly, the administration of each *B. pseudolongum* strain resulted in major divergence in the structure, composition, and function of gut microbiota. This was accompanied by markedly different changes in intestinal transcriptional activities, suggesting strain-specific modulation of the endogenous gut microbiota as a key to immune modulatory host responses. Our study demonstrated a single probiotic strain can influence local, regional, and systemic immunity through both innate and adaptive pathways in a strain-specific manner. It highlights the importance to investigate both the endogenous gut microbiome and the intestinal responses in response to probiotic supplementation, which underpins the mechanisms through which the probiotic strains drive the strain-specific effect to impact health outcomes.

## Introduction

*Bifidobacterium* spp. are naturally occurring residents within the gastrointestinal (GI) tract of mammals and are typically considered beneficial^1,2^. Due to their purported health-promoting properties, *Bifidobacterium* spp. have been incorporated into many live biotherapeutic (LBP) prophylactic formulations, mostly known for applications in alleviating intestinal inflammatory conditions^3–7^. The potential mechanisms underlying the health benefits of *Bifidobacterium* include the suppression of gut pathogens growth^8,9^, capabilities to alter gut metabolism and to enhance epithelial barrier function^10,11^, and anti-inflammatory modulation of host immunity^12–15^. In particular, their immunomodulatory properties are not limited to the direct effects on GI tissues, but also indirect effects enacted through their influence on the gut microbiota^16^. *Bifidobacterium* spp. are known to participate in mutualistic interactions with endogenous intestinal microorganisms that can subsequently evoke both immediate as well as delayed immune responses^17,18^. However, the cellular and molecular underpinnings *Bifidobacterium*’s biotherapeutic effects remain unclear with contradictory findings reported^19^. Fundamentally important questions such as what specific mechanisms through which they exert immunomodulatory effects, to what extent the interactions with the gut microorganisms affect the immune responses, and what are the roles of elicited intestinal responses in these processes remain outstanding.

The immunomodulatory properties of individual *Bifidobacterium* spp. are strain-dependent, despite similar effects produced by closely related strains (i.e., alleviation of lactose intolerance or improved host antimicrobial activity)^20–22^. In fact, immunomodulatory effects are independent of microbial phylogeny^20^. Recent investigations suggested that differences in cell wall composition and structure might be responsible for strain-specific immunomodulatory effects^23^. Microorganism-associated molecular patterns (MAMPs) possess variable biochemistry, even between strains, serving as microbial stimuli that orchestrate molecular cascades in the host immune response and mucosal homeostasis^24–26^. Exopolysaccharide (EPS) and pili may play a role in *Bifidobacterium’s* strain-specific pro-homeostatic immune modulation^27,28^. Other molecular mechanisms such as lipoteichoic acid and specific metabolites such as acetate could also contribute to strain-specific immunity^26,29,30^. Comparisons of the immunomodulatory properties of closely related strains can be leveraged to identify which are strain-specific and to characterize the microbial determinants of specific host responses, which will provide the basis to rationally hone biotherapeutics for prophylactic applications^15,31,32^.

We previously showed, using a major histocompatibility complex (MHC)-mismatched murine cardiac transplant model, that fecal microbiota transfer (FMT) caused shifts in the gut microbiota which profoundly influenced allograft outcomes^33^. FMT using stool samples from healthy pregnant mice (immune suppressed) resulted in improved long-term allograft survival and prevented inflammation and fibrosis in grafts, as compared to FMT using stool samples from colitic or nonpregnant control mice^33^. *B. pseudolongum* was revealed as a microbial biomarker for the pregnant mouse gut microbiota, from which we subsequently isolated and sequenced as UMB-MBP-01^34^. Importantly, gavage with UMB-MBP-01 alone reproduced the same improved graft outcomes as FMT using whole stool of pregnant mice, implicating this strain as one of the main responsible microbes^33^. Thus, the murine tropic strain UMB-MBP-01 may serve as a model organism to investigate the mechanisms of microbe-driven immunomodulation.

In this study, we used a combination of cell culture and in vivo experimentation with mice, and comparative genomics approaches to investigate the mechanisms underpinning the strain-specific immunomodulatory capabilities of probiotic *Bifidobacterium* strains. We performed a genome-wide comparison of UMB-MBP-01 to all other *B. pseudolongum* genomes, including three additional *B. pseudolongum* strains (E, EM10, EM13) isolated from the same feces sample of a pregnant mouse, as well as to the porcine tropic strain ATCC25526, in order to investigate the genetic attributes underlying their immunomodulatory properties. Further, we revealed distinct effects on local and systemic immunity induced by UMB-MBP-01 and ATCC25526, using both cell culture and in vivo approaches. Importantly, the oral administration of the two *B. pseudolongum* strains resulted in profound alterations in composition, structure and function of the murine gut microbiome, accompanied with markedly different intestinal transcriptome activities. These observations indicate that a single probiotic strain can influence local, regional, and systemic immunity through both innate and adaptive pathways in a strain-specific manner. Our study suggests that the modulation of the endogenous gut microbiome is a key element by which *Bifidobacterium* probiotic strains impose their immunomodulatory effects. A deeper understanding of the strain specificity and mechanisms of action through which specific strains regulate host responses will facilitate the clinical translation of live therapeutics and the development of potential targets for immunomodulatory therapy.

## Results

### Differential activation and cytokine responses in dendritic cells and macrophages induced by B. pseudolongum strains ATCC25526 and UMB-MBP-01

**Figure 1:**
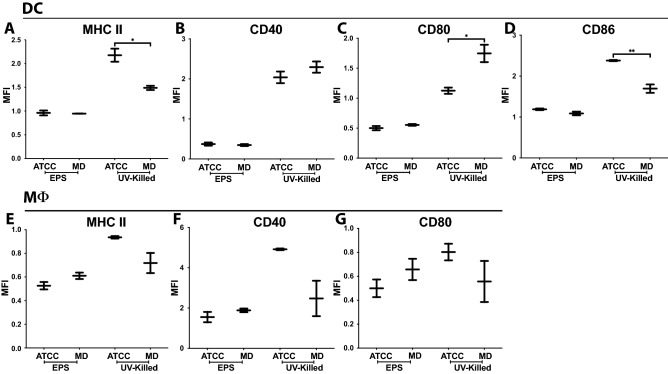
*Bifidobacterium* alters DC and MΦ surface phenotype. DC and MΦ cultured with media alone (to which treatment groups are normalized), ATCC25526 (ATCC) or UMB-MBP-01 (MD) UV-killed *Bifidobacterium* or EPS derived from each strain. After 24 h of culture, cells analyzed by flow cytometry. DC gated on live CD11c + , and MΦ gated on live F4/80 + populations. DC stained for (**A**) MHC class II, (**B**) CD40, (**C**) CD80, and (**D**) CD86. MΦ stained for (**E**) MHC class II, (**F**) CD40 and (**G**) CD80. MFI: normalized mean fluorescence intensity; MFI values normalized to control and compared using one-way ANOVA. *p value < 0.05; **p value < 0.01. UV-killed MΦ data representative of 3 separate experiments (one of which is shown), 2 wells/culture condition, i.e. 6 total wells per condition over 3 experiments. EPS MΦ data representative of 2 separate experiments (one of which is shown), 2 wells/culture condition, i.e. 4 total wells per condition over 2 experiments. DC data merged from one experiment with EPS treatment and 2 experiments with UV-killed bacteria (one of which is shown), each data set is normalized to its respective “media only” control, i.e. 4 total wells per condition over 2 experiments for UV-killed bacteria and 2 total wells per condition for EPS. The raw files from these experiments are available to download at https://doi.org/10.6084/m9.figshare.21685814.

To understand the immunomodulatory impact of the two *B. pseudolongum* strains, bone marrow derived dendritic cells (BMDC) and peritoneal macrophages (MΦ) were treated with UV-killed bacteria or isolated *Bifidobacterium* exopolysaccharide (EPS). We first examined the effect of these treatments on expression of myeloid costimulatory receptors using flow cytometry to assess whether contact with whole bacteria or simply bacterial surface components were necessary for immunomodulation. For BDMCs, treatment with either *B. pseudolongum* strain stimulated increased CD40 and CD86 expression, however, CD86 expression was greatest after treatment with ATCC25526 UV-killed bacteria compared to UMB-MBP-01 (Fig. 1A–D). Treatment with ATCC25526 UV-killed bacteria stimulated increased BDMC MHC class II, while treatment with UMB-MBP-01 stimulated increased CD80 expression. Neither UMB-MBP-01 EPS nor ATCC25526 EPS altered expression of these surface receptors on BMDCs. The MΦ cell surface receptors were not differentially affected by treatment with UV-killed bacteria or EPS (Fig. 1E–G). Overall, ATCC25526 and UMB-MBP-01 UV-killed bacteria, but not their respective EPS, each triggered a unique activation of important costimulatory receptors on innate myeloid cells in culture.

**Figure 2:**
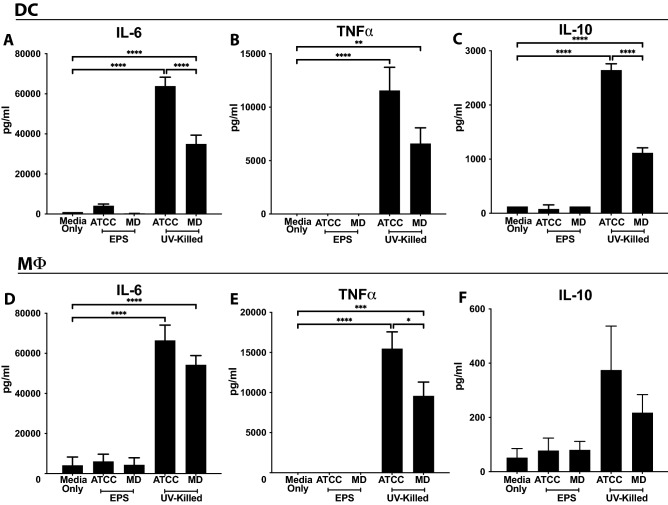
*Bifidobacterium* alters DC and MΦ cytokine secretion. DCs (**A–C**) or MΦ (**D–F**) stimulated with EPS or UV-killed ATCC25526 (ATCC) or UMB-MBP-01 (MD), and 24 h later supernatants analyzed for (**A**, **D**) IL-6, (**B**, **E**) TNFα, and (**C**, **F**) IL-10 by ELISA. Treatments compared using one-way ANOVA. * p value < 0.05; **p value < 0.01, ***p value < 0.001, ****p value < 0.0001. UV-killed bacteria data representative of 3 separate experiments, 2–3 wells/culture condition, 2 technical replicates/well (supernatants from each well split and analyzed in duplicate), i.e. 4–6 wells per condition per experiment, 14 total wells per condition over 3 experiments. EPS data representative of 2 separate experiments, 2–3 wells/culture condition, 2 technical replicates/well (supernatants from each well split and analyzed in duplicate), i.e. 4 wells per condition per experiment, 10 total wells per condition over 2 experiments. The raw files from these experiments are available at https://doi.org/10.6084/m9.figshare.21685838.

We next examined the effect of UV-killed bacteria and EPS alone on cytokine production in innate immune cells. Using ELISA, both BMDC and MΦ showed increased secretion of IL-6, TNFα, and IL-10 when stimulated with UMB-MBP-01 or ATCC25526 UV-killed bacteria. Induction of cytokine expression was also strain-specific as ATCC25526 UV-killed bacteria stimulated a greater increase in IL-6 and IL-10 than UMB-MBP-01 in BMDCs (Fig. 2A, C), suggesting a more pro-inflammatory effect. TNFα expression was also increased to a greater extent by ATCC25526 compared to UMB-MBP-01 with a borderline statistical significance (*p* = 0.059, Fig. 2B). For MΦ, treatment with ATCC25526 UV-killed bacteria increased TNFα compared to UMB-MBP-01 (Fig. 2E), suggesting a greater pro-inflammatory effect in MΦ as well as BMDC, whereas there were no strain-specific differences in IL-6 or IL-10 (Fig. 2D, F). EPS of either *B. pseudolongum* strains did not stimulate cytokine production in either BMDC or MΦ. Similar to co-stimulatory receptor activation, UMB-MBP-01 and ATCC25526 UV-killed bacteria elicited unique myeloid cell cytokine responses that differed from one another and were not recapitulated by EPS.

### Bifidobacterium strains induce distinct changes in local and systemic leukocyte distribution and lymph node morphology

**Figure 3:**
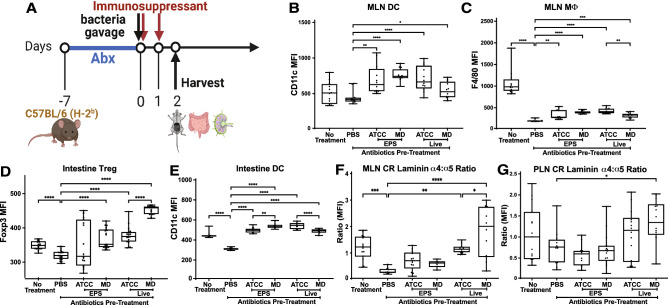
*Bifidobacterium* strains induce unique changes in local and systemic immune cell distribution and LN architecture. (**A**) Experimental design. C57BL/6 mice treated with antibiotics for 6 days followed by oral gavage with *B. pseudolongum* ATCC25526 (ATCC), UMB-MBP-01 (MD), or PBS (control). Mice then treated with the immunosuppressant tacrolimus for the next two days. Tissues harvested 2 days after oral gavage of bacteria. Frozen MLN sections stained for (**B**) CD11c + DC, and (**C**) F4/80 + MΦ. Small intestine stained for (**D**) Foxp3 + Treg, and (**E**) CD11c + DC. LN stained for laminin α4 and laminin α5 with their ratio depicted for (**F**) MLN CR, and (**G**) PLN CR. MFI values normalized using the sum of mean, and categories compared using one-way ANOVA. *p value < 0.05; **p value < 0.01, ***p value < 0.001, ****p value < 0.0001. Data representative of 2 separate experiments, 3 mice/group, i.e. 6 total mice per condition over 2 experiments. The raw files from these experiments are available at https://doi.org/10.6084/m9.figshare.21685850.

We next assessed whether *Bifidobacterium* strains differentially induced changes in immune cell distribution and lymph node (LN) architecture in vivo using a mouse model. Mice received broad spectrum antibiotics for 6 days, a regimen that depleted endogenous microbiota^35^, followed by oral gavage with live bacteria (bacteria gavage) or their EPS only, and then daily immunosuppression with tacrolimus (3 mg/kg/d subcutaneously) (experiment design in Fig. 3A). Tacrolimus is the most commonly used clinical immunosuppressive drug and was thus used to recapitulate the immunologic variables to which transplant recipients are subjected, as demonstrated in our clinically relevant murine model to study bacteria-driven allograft immunomodulation^33^. Two days after gavage, mesenteric and peripheral LNs (MLN and PLN) and intestinal tissues were harvested.

The effect of these microbiota on the distribution of immune cell populations was assessed by flow cytometry to characterize overall LN cell content, and immunohistochemistry (IHC) to characterize relative immune cell positioning and architectural changes in LN and intestinal segments. Using flow cytometry (gating protocol in Supplemental Fig. 3), we observed decreased MLN Foxp3 + regulatory T cells (Treg) in UMB-MBP-01 EPS treated animals, but otherwise no other differences in the number and proportion of innate myeloid (DC, MΦ) or adaptive lymphoid cells (CD4 T cells, CD8 T cells, Treg, and B cells) in the MLN or PLN of mice with *B. pseudolongum* gavage compared to those treated with antibiotics alone or untreated controls (Supplemental Fig. 4A–L). We therein used IHC to investigate changes in cell content versus microanatomic shifts in cell positioning and interactions, as our previous work showed that architectural and cellular changes within the LN cortical zone were most critical in mediating immune tolerance and suppression^36,37^.

Using IHC to examine the MLN and PLN T cell cortex, neither bacteria gavage nor EPS affected the number of Treg present. This contrasts with the flow cytometry results above which showed that UMB-MBP-01 EPS treatment caused a decrease in MLN Treg (Supplemental Fig. 4D). Gavage with live bacteria and EPS of both strains increased DCs in MLN compared to control (Fig. 3B), but not PLN (Supplemental Fig. 4M). The number of DCs enumerated by flow cytometry, however, did not change in MLN or PLN (Supplemental Fig. 4E, K). MΦ increased in the cortex of MLN after bacteria gavage or EPS treatments from both strains (Fig. 3C), but not in PLN (Supplemental Fig. 4N). ATCC25526 bacteria gavage also resulted in a greater increase in MLN MΦ compared to UMB-MBP-01 (Fig. 3C). Again, this result contrasts with flow cytometry data where there was no difference in MΦ populations in MLN or PLN after treatment. The differences between the flow cytometry and histologic results for Treg are likely due to the focus of histologic analysis only on cells in the cortex while flow cytometry summates all the cells in the entire LN, emphasizing that microanatomic shifts in cell positioning are more outstanding than cell content.

**Figure 4:**
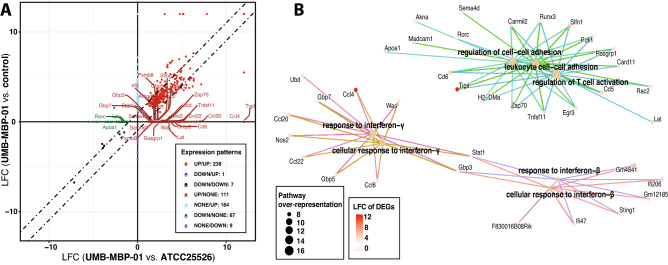
Transcriptome profiling of intestinal tissues in response to ATCC25526 or UMB-MBP-01. (**A**) Quadrant plot to show whether differential expressed genes (DEGs) have the same or opposite relationships between each of the pairwise comparison of UMB-MBP-001 vs control and UMB-MBP-001 vs ATCC25526. DEGs were determined using log2 fold change (LFC) > (+ /−)1 and false discovery rate (FDR) < 0.05. (**B**) Gene-Concept network for most over-represented Gene Ontology (GO) terms to depict over-represented functions based on q-value and gene-count. Over-representation analyses^106^ of DEGs that are only different abundant in UMB-MBP-001 vs control but not in ATCC25526 vs control, using GO ontologies performed using enrichGO function of clusterProfile Bioconductor package^107^. For pairwise comparison enrichment analyses for any two conditions, please refer to Supplemental Fig. 4.

We next assessed LN architecture using the ratio of laminin α4 to laminin α5 in the LN T cell cortex of the cortical ridge (CR) and around the high endothelial venules (HEV) by IHC. LN stromal fiber structures are important mediators of immune responses^36^, and an increased laminin α4: α5 ratio is indicative of immune tolerance and suppression^38^. In the MLN CR, both UMB-MBP-01 and ATCC25526 bacterial gavage increased the laminin α4: α5 ratio, with UMB-MBP-01 causing an even greater increase (Fig. 3F). The laminin α4: α5 ratio was not changed around the MLN HEV by either bacteria gavage or EPS (Supplemental Fig. 4O). In PLN CR, only UMB-MBP-01 bacteria gavage resulted in an increased laminin α4: α5 ratio (Fig. 3G). Overall, gavage with both *B. pseudolongum* strains increased local MLN CR laminin α4: α5 ratios, while only UMB-MBP-01 increased the laminin α4: α5 ratio in systemic PLN CR. Increased MLN CR laminin α4: α5 ratios were also more prominent with UMB-MBP-01 compared to ATCC25526*,* further demonstrating strain-specific differences in immune modulation.

Only IHC was employed to examine the small intestinal segments, since dissociation of this organ followed by leukocyte isolation results in major losses of total cells and unequal loss of cell subsets compared to LN where dissociation is far easier^39^. Both UMB-MBP-01 and ATCC25526 bacteria gavage or EPS resulted in significantly more Treg compared to PBS control, while UMB-MBP-01 resulted in even more Treg compared to ATCC25526 (Fig. 3D), emphasizing its pronounced impact on intestinal Treg. ATCC25526 and UMB-MBP-01 bacteria gavage resulted in more DC compared to no bacteria control, while ATCC25526 also resulted in more DC compared to UMB-MBP-01 (Fig. 3E). UMB-MBP-01 and ATCC25526 EPS resulted in increased DC compared to PBS control, while UMB-MBP-01 EPS also resulted in more DC compared to ATCC25526 EPS (Fig. 3E). Intestinal MΦ did not significantly change after bacteria or EPS gavage. In contrast with our findings in cell culture where EPS was generally inactive, in vivo treatment with EPS alone stimulated similar innate myeloid cell and Treg increases in gut and MLN compared to increases induced by bacterial gavage. Overall, gavage with live bacteria and EPS altered gut associated innate myeloid cells and Tregs without affecting systemic distribution, as evidenced by unchanged PLN populations by flow and IHC (Supplemental Fig. 3J–N). The gavage of live UMB-MBP-01 bacteria was most impactful on intestinal Treg, while ATCC25526 most pronouncedly affected DC. Again, these results indicate both shared characteristics and some significant differences between the immunomodulatory effects of the two different *B. pseudolongum* strains on intestinal segments.

### Markedly different intestinal transcriptional activities in response to UMB-MBP-01 than to ATCC25526

To determine the effect of UMB-MBP-01 and ATCC25526 on host gene expression, we characterized the transcriptome of mouse intestinal tissues harvested two days after gavage with either UMB-MBP-01, ATCC25526, or no bacteria control. Differentially expressed genes (DEGs) were identified by comparing the two treatment groups to the control and revealed both shared and strain-specific effects on transcription (Supplemental Table 6B-D). A total of 420 and 425 DEGs were observed in comparisons of UMB-MBP-01 vs. control and ATCC25526 vs. control, respectively, and 139 DEGs were observed comparing UMB-MBP-01 to ATCC25526 directly. Based on the log_2_ fold change (LFC) scale of DEGs, the strongest intestinal response was elicited by UMB-MBP-01, compared to either ATCC25526 or control (Supplemental Fig. 5). Functional enrichment analyses revealed the effects elicited by UMB-MBP-01 were mainly involved in positive regulation of cell activation, leukocyte and lymphocyte activation, B cell activation, and somatic recombination of immunoglobulin superfamily domains (Supplemental Fig. 5A,C). ATCC25526 elicited responses in phagocytosis, membrane invagination, defense responses to bacterium, and complement activation (Supplemental Fig. 5E). These results further support our observations that ATCC25526 elicited distinct host responses compared to UMB-MBP-01, which induced greater numbers of DEGs and stronger host responses.

**Figure 5:**
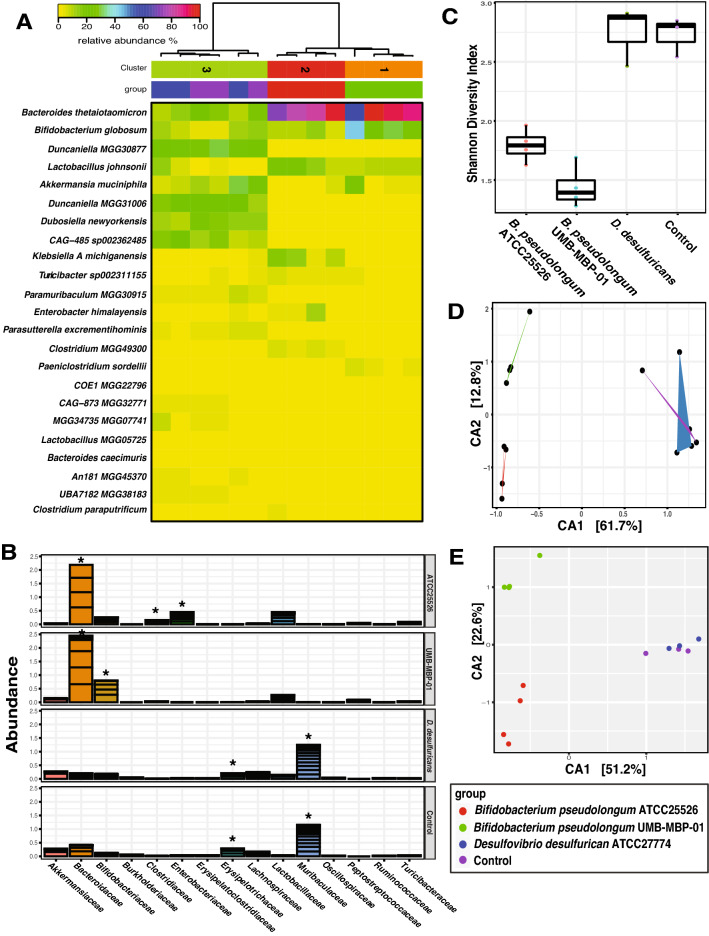
Alterations in gut microbiome after bacterial gavage. (**A**) Heatmap of the top most abundant intestinal bacterial taxa relative abundance in mice intraluminal samples. Ward linkage clustering based on Jensen-Shannon distance was calculated using the vegan package in R^101^. Taxonomic profiles of the microbial community were characterized using the comprehensive mouse gut metagenome (CMGM) catalog^40^. R codes and input dataset used to generate the heatmap was deposited in github (https://github.com/igsbma/genome_paper). (**B**) Cumulative relative abundance of major bacterial families. The relative abundances of each family are stacked in order from greatest to least, and are separated by a horizontal line. (**C**) Shannon diversity index (within-community diversity) of the four experimental groups. Canonical Correspondence Analysis (CCA) of (**D**) microbial functional pathways and (**E**) taxonomy. Pathways were characterized using HUMAnN2 (v0.11.2)^98^ and Uniref90 database^97^ based on Bray–Curtis distance. CA1 and CA2 selected as the major components based on the eigenvalue.

We further examined the host responses to both UMB-MBP-01 and ATCC25526 as well as those respond only to one but not the other, to pinpoint the differential host responses induced by the two strains. Of the DEGs identified comparing UMB-MBP-01 or ATCC25526 to the control (n = 411 and 416), 59.6% and 58.9%, respectively, were identified in both comparisons (Fig. 4A, Supplemental Table 6). These overlapping DEGs (N = 238) and the condition-specific DEGs, that included 164 DEGs only up-regulated in UMB-MBP-01 versus control and 111 DEGs only upregulated in UMB-MBP-01 versus ATCC25526, comprised the majority of all DEGs (85.9%). Downregulated genes accounted for only a small fraction of all DEGs (14.1%) and the majority of them were identified only in the comparison of UMB-MBP-01 versus ATCC25526 (N = 67, 80.1% of downregulated). The DEGs that were upregulated in both UMB-MBP-01 vs. control and ATCC25526 vs. control include B cell immunity, collagen metabolism, immunoglobulin protein expression, cytokines (IL-1β, IL-10, IL-13, IL-21), TNF receptor superfamily, among others (Supplemental Table 6). Two functional pathways were enriched in UMB-MBP-01 vs control, but not in ATCC25526 vs control: regulation of cell–cell adhesion, regulation of T cell activation, and the response to interferon γ and interferon β (Fig. 4B). In contrast, the host responses to ATCC25526 but not UMB-MBP-01 were enriched in functions involved in fatty acid metabolism, lipid localization, acylglycerol metabolism, and cholesterol and sterol homeostasis (Supplemental Fig. 5D). Together these data further indicated that the effects of UMB-MBP-01 or ATCC25526 were mediated through different pathways. The ATCC25526 strain appeared to exert immunomodulatory effects, at least in part, via stimulation of phagocytosis and induced lipid metabolism, while the UMB-MBP-01 strain exerted stronger effects, mostly through upregulating antibody secretion and regulation of multiple aspects of lymphocyte function, including cytokines, adhesion, and activation.

As extracellular molecules may play an important role in eliciting immunomodulatory effects, we also compared intestinal gene expression following gavage with live *B. pseudolongum* bacteria to that with *B. pseudolongum* derived EPS. The comparison revealed DEGs that were mostly group-specific without much overlap with EPS vs. control group (12.1%, N = 39) (Supplemental Fig. 6A,B). These data indicated that the predominant intestinal transcriptional responses were due to *B. pseudolongum* bacteria gavage (62.6%, N = 201), compared to DEGs in EPS gavage (25.5%, N = 82). Gene-pathway network analyses indicated B cell receptor activation and signaling, antigen-receptor mediated signaling, and phagocytosis recognition and engulfment were highly upregulated by *B. pseudolongum* bacteria gavage (Supplemental Fig. 6C). While both live bacteria and EPS induced antimicrobial circulating immunoglobulin expression, the transcriptional effects were an order of magnitude higher for live bacteria (Supplemental Fig. 6C,D). This result was commensurate with the observations above that EPS did not stimulate cytokine production or cell surface costimulatory receptor expression in either BMDC or MΦ in cell culture. Together, our data support the speculation that the immunomodulatory effects of *B. pseudolongum* are mostly potentiated through pathways such as influencing gut microbiomes and host metabolic activities, secreted molecules, and/or other cell membrane components, while the surface structure of EPS may play a minor role in these biological processes.

### Different B. pseudolongum strains elicit rapid, profound alterations in both structure and function of gut microbiome

We next investigated the impact of bacterial gavage on the gut microbiome using shotgun metagenomic sequencing of the intraluminal fecal content (40.6 ± 7.7 million reads per sample; Supplemental Table 2B). Taxonomic composition was established using the comprehensive mouse gut metagenome catalog (CMGM)^40^ designed specifically to characterize the mouse gut microbiome (Fig. 5A, Supplemental Table 7). A significant reduction in gut microbial community diversity was observed after both UMB-MBP-01 and ATCC25526 gavage, with UMB-MBP-01 gavage resulting in the lowest diversity (Fig. 5C). After *B. pseudolongum* administration, the most outstanding changes in specific taxonomic groups were the marked increases in the relative abundance of *Bacteroides thetaiotaomicron* and *Lactobacillus johnsonii* and relative depletion of *Muribaculaceae* and *Erysipelotrichaceae* (Fig. 5B, Supplemental Fig. 7). Gavage with the *Desulfovibrio* did not produce a significant change in the microbiome, and *Muribaculaceae*, *Erysipelotrichaceae*, and *Lachnospiraceae* were the most abundant groups in these communities and in the not-treatment control communities. These data indicated the gavage of either *B. pseudolongum* strains profoundly altered the gut microbial community, with a significant reduction in the relative abundance of endogenous gut microorganisms.

Canonical Correspondence Analysis (CCA) on both community taxonomic profiles and functional pathways resulted in concordant clustering patterns, in that ATCC25526 or UMB-MBP-01 each resulted in a distinct community, and that were clearly separate from *Desulfovibrio*-treated and no bacteria controls (Fig. 5D,E). Based on linear discriminant analysis (LDA) effect size (LEfSe) analysis^41^, UMB-MBP-01 resulted in a significantly higher relative abundance of *B. pseudolongum* than ATCC25526 (19.8 ± 6.1% vs. 6.1% ± 2.0%, *P* = 0.02, Supplemental Fig. 8A,B). This suggests that murine isolate strain UMB-MBP-01 was better able to colonize the mouse gut than the porcine isolate strain ATCC25526 in the murine gut microenvironment. This is not surprising given the provenance of the UMB-MBP-01 strain and its probable prior adaptation to the murine gut microenvironment that it was originally derived from, comparing to ATCC25526 and *Desulfovibrio* that have different host origins. On the other hand, *Enterobacteriaceae* (*Klebsiella michiganensis* and *Enterobacter himalayensis*)*,* and *Clostridiaceae* (*Clostridium paraputrificum* and *Clostridium* MGG49300) were significantly more enriched in terms of relative abundance in the ATCC25526 treated group but were mostly absent in the UMB-MBP-01 gavage mice (Supplemental Fig. 8C–F). Based the scaled eigenvalue, the top taxa and pathways that contributed to the separation of the clusters in ordination analyses were identified (Supplemental Fig. 9A,B, Supplemental Table 9). The ATCC25526 cluster was attributed to *Enterobacteriaceae* and *Clostridiaceae*, and the top contributors included *K. pneumonia*, *K. michiganensis*, *B. animalis*, *E. himalayensis*, and *Clostridium paraputrificum*. The most prominent pathways attributed to ATCC25526 cluster include motility (peptidoglycan maturation), gluconeogenesis, energy conversion (fatty acid β-oxidation), and L-threonine biosynthesis. On the other hand, *Akkermansia muciniphila*, *Paeniclostridium sordellii*, and *B. pseudolongum* were among the top significant contributors to the UMB-MBP-01 cluster. The most outstanding pathways for UMB-MBP-01 included ribonucleotide and amino acid biosynthesis (folate transformation, L-isoleucine, L-arginine, L-lysine) and pyruvate fermentation (pyruvate/acetyl-CoA pathway). Together, the data indicated UMB-MBP-01 or ATCC25526 each altered the gut microbiota profoundly and distinctively, which may contribute to their distinct immunomodulatory effect.

### High genome plasticity of B. pseudolongum reflects strong host adaptability

The pangenome of *B. pseudolongum* was constructed using 79 strains including the 4 strains sequenced as part of this study (Supplemental Table 1A). Homologous gene clusters (HGCs) were identified in this set of genomes based on all-versus-all sequence similarity (Supplemental Table 1B). A total of 4,321 *B. pseudolongum* HGCs were revealed, among which 31.7% were core (present in almost all strains), 57.0% were dispensable (singleton or present in very few genomes), and the remaining 11.3% were considered accessory. *B. pseudolongum* demonstrated a smaller pangenome size that was 87.8% of *B. breve* and 59.5% of *B. longum* pangenomes (Supplemental Fig. 1). *B. pseudolongum* had the fewest number of conserved HGCs (N = 1370) but the largest proportion of dispensable pangenome (57.0%) compared to the two other *Bifidobacterium* species *B. longum* and *B. breve* that were both human associated. This disproportionally large dispensable pangenome may be indicative of strong niche adaptation capabilities of *B. pseudolongum*, reflecting its broad host range, being widely distributed among mammals^42^.

Whole genome sequencing was performed on three *B. pseudolongum* strains (E, EM10, and EM13) isolated from the same pregnant mice feces as UMB-MBP-01 (sequencing statistics in Supplemental Table 2A). Comparison among the four murine strains revealed 1,520 shared coding DNA sequence (CDS), which comprised 97.2% of UMB-MBP-01 coding genes (Supplemental Table 1C). 107 CDS were conserved in at least two but not in all four genomes, and 37 CDS were strain-specific. Most of these genes had unknown functions, and those with known functions related to bacteriophage assembly and function (i.e., capsid protein, integrase, transposes, bacteriophage replication gene, cell lysis protein, microvirus H protein) or carbohydrate hydrolysis and transport (glycosyl hydrolases, ABC transporter permease). On the other hand, comparison between UMB-MBP-01 and ATCC25526 revealed 1,351 shared CDS (86.4% of UMB-MBP-01 coding genes), and 157 genes that belonged to one strain but not the other (Supplemental Table 1D). Interestingly, most of these strain-specific genes also belonged to the categories of bacteriophage assembly and functions as well as carbohydrate hydrolysis and transport, in addition to genes with unknown function. Together these data suggested bacteriophage-mediated transduction might have been a major contributor to dissemination of carbohydrate metabolism capabilities, potentially through horizontal gene transfer among closely related murine-derived strains, as well as more distantly related *B. pseudolongum* strains.

**Figure 6:**
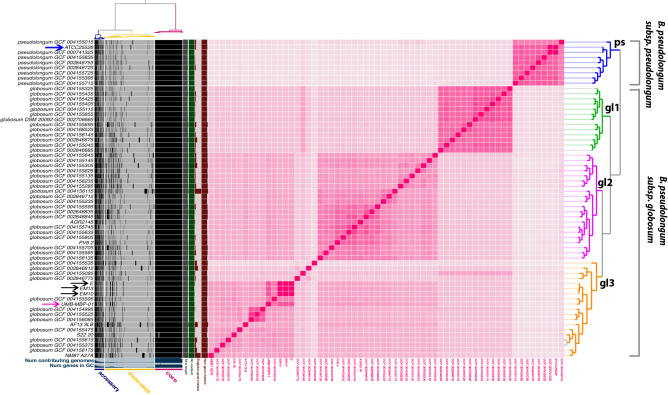
Pangenome analyses of *B. pseudolongum* genomes. Pangenome constructed using 79 strains, including the 5 strains sequenced in this study (Supplemental Table 3) and displayed using anvi’o *vers* 6.2^74^. Homologous gene clusters (HGCs) were identified based on all-versus-all sequence similarity in left panel and categorized as core, accessory or dispensable depending on their level of conservation. Genome ANI (Average Nucleotide Identity) was calculated using Sourmash vers 3.3^76^. Blue arrows indicate the two strains compared: ATCC25526 and UMB-MBP-001. Black arrows indicate the other three *B. pseudolongum* strains isolated from the source stool of pregnant mice.

Whole genome Average Nucleotide Identity (ANI) clustering suggested two subspecies, *B. pseudolongum* subsp. *pseudolongum* clade that contained ATCC25526, and *B. pseudolongum* subsp. *globosum* clusters that had three distinct clades I-III (Fig. 6). Subspecies *globosum* clade III had the largest number of coding genes (1642 ± 70) among all clades and contained UMB-MBP-01 and the three isolates from the source stools of pregnant mice. The subspecies *pseudolongum* clade had the smallest number of coding genes among all clades (1519 ± 35.6). Overall, 1599 HGCs accounted for 37.0% of *B. pseudolongum* pangenome were identified as clade-specific (> 90% genes belonging to the same clade), and the majority originated from *globosum* clade III (N = 648), while clade *pseudolongum* provided the fewest (N = 130). The large number of clade-specific genes found in *globosum* clade III genomes suggested a high degree of genome plasticity to facilitate adaptation to cope with environmental heterogeneity. Further functional enrichment analyses revealed *globosum* cluster III-specific HGCs were mostly involved in periplasmic transport systems, permeases and glycoside hydrolases (GHs), particularly the families GH29 (α-L-fucosidase), GH3 (β-glucosidase) and GH31 (α-glucosidase) (Supplemental Table 1E). No GH families were enriched in any of the other clades. Together, UMB-MBP-01 and ATCC25526 belonged to two different subspecies, each of which comprises considerable genetic variation. The genome of UMB-MBP-01 contained more clade-specific genes and was enriched for genetic features in carbohydrate metabolism to assimilate greater varieties of glycans. Further investigation is needed to characterize the role of carbohydrate metabolism in niche adaptive capabilities of murine isolates in the glycan-rich murine gut microenvironment.

We sought to characterize the secretome of *B. pseudolongum* by in silico examining protein localization based on the presence of a signal peptide^43^. Proteins which are secreted extracellularly have the potential to directly interact with the other gut microorganisms and with host tissues (Supplemental Table 3A)^27,44^. Overall, the sec-dependent secretion machinery, but not the twin-arginine (Tat) system, was conserved in all *B. pseudolongum* strains, indicating protein translocation function was conserved but likely occurs only in the unfolded state^45^. Secreted proteins were more likely to be part of the dispensable genome (73% of secreted proteins versus 53% of cytoplasmic proteins; Supplemental Table 3B), indicating a high degree of diversity in the secretome among strains of *B. pseudolongum*. Proteins which were predicted to be extracellularly secreted include solute-binding proteins of ABC transporter systems, amidases related to the peptidoglycan hydrolysis, glycosyl hydrolyses, cell surface proteins that make up pilus subunits, and cell wall-degrading peptidases. Interestingly, the secretome of the clade containing ATCC25526 was enriched for collagen adhesion proteins (Supplemental Table 3C) but lacked multiple secreted GH25 extracellular proteins. These proteins are prevalent in the clade which includes UMB-MBP-01 and are involved in the binding and hydrolysis of peptidoglycan (Supplemental Table 5D). As peptidoglycan components were implicated in important aspects of mucosal immunological signaling^46^, this may contribute to varied immunomodulatory capabilities between UMB-MBP-01 and ATCC25526.

### Specialized carbohydrate metabolizing capabilities of UMB-MBP-01 and ATCC25526

The abundance of *Bifidobacterium* glycolytic features is reflective of their metabolic adaptation to the complex carbohydrate-rich GI tract^47,48^. We performed in silico prediction of the carbohydrate fermentation capabilities to comprehensively investigate glycan-assimilation capabilities for all 79 *B. pseudolongum* genomes, using with the Carbohydrate-Active enZYmes Database (CAZy) database^49^. This analysis revealed 236 genes of *B. pseudolongum* pangenome encoding predicted carbohydrate-active enzymes from 34 glycosyl hydrolase families, 14 glycosyl transferase families and eight carbohydrate esterase families (Supplemental Table 4A). Only 33.5% of the carbohydrate-active enzyme coding genes belonged to the core pangenome. Core GHs included those mostly responsible for the breakdown of plant-derived polysaccharides (*i.e.*, starch) and a wide range of other carbohydrates, such as GH13 (glycosidase), GH77 (α-amylase), GH43 (β-xylosidase), GH36 (α-galactosidase), GH2 (β-galactosidase), GH3, and GH6 (cellobiohydrolases). Notably, GH13 is the enzyme family known to be most commonly found in *Bifidobacterium* genomes and active on a wide range of carbohydrates including the plant-derived starch and the related substrates of trehalose, stachyose, raffinose, and melibiose^48,50^. Conversely, 47.9% of the identified carbohydrate-active enzymes genes were found in the dispensable pangenome. The *globosum* clade II (N = 72) and III (N = 58) encoded most of these enzymes, while the *pseudolongum* clade encoded the least (N = 13). These results demonstrated the highly specialized carbohydrate assimilation gene repertoires of different strains, particularly in *globosum* clade II and III.

Using UMB-MBP-01 as the reference for all other *B. pseudolongum* strains, both conserved and specific glycohydrolases capabilities were revealed (Supplemental Fig. 2, Supplemental Table 1F, 4B). Interestingly, the clusters based on GH are mostly in agreement with the clades generated based on ANI, suggesting distinct carbohydrates assimilation capabilities of different *B. pseudolongum* clades. GH29, GH31, GH42 (β-galactosidase), and ABC-type polysaccharide transport permease genes were most prevalent in *globosum* clade III that contained UMB-MBP-01. Further, GH36, GH2, and GH94 (cellobiose phosphorylase) were found absent in subspecies *pseudolongum* clade but prevalent in *globosum* clade III. In particular, an uncommon GH23 family (peptidoglycan lyses) was only observed in UMB-MBP-01 and the three other isolates from the pregnant mouse. Overall UMB-MBP-01 and ATCC25526 share some enzymatic capabilities in metabolizing dietary polysaccharides and host-derived glycogens, while also having specialized glycohydrolases genes.

We further characterize the carbohydrate utilization capabilities of UMB-MBP-01 and ATCC25526 using anaerobic microplates pre-coated with various carbon sources. Out of the 95 carbon sources tested, the two strains demonstrated the same capabilities on 86 (90.5%) (Supplemental Table 5), including key carbon sources N-acetyl-D-glucosamine, D-fructose, L-fucose, α-D-glucose, glucose-6-phosphate, maltose, maltotriose, D-mannose, D-sorbitol, and pyruvic acid. Two relatively uncommon sugars D-melibiose and D-raffinose could be metabolized by ATCC25526 but not UMB-MBP-01. On the other hand, D-galactose, D-gluconic acid, D-glucosaminic acid, glycerol, D-mannitol, α-ketovaleric acid, and D, L-lactic acid were uniquely metabolized by UMB-MBP-01. This result is in principle in an agreement of the specific GH families predicated in silico. Together these data indicated a wide range of carbohydrate metabolizing capabilities ranging from dietary to host-derived glycans for both strains, while UMB-MBP-01 had specialized capabilities to metabolize galacto-oligosaccharides.

## Discussion

*Bifidobacterium pseudolongum* demonstrates great intraspecies genetic diversity and shows patterns consistent with host specificity, rendering it an advantageous model organism to study the effect of intraspecies variation on host immunomodulation^42^. Further, as a predominant species in the murine GI tract, *B. pseudolongum* displays an extensive enzymatic capacity and might act as a keystone species in this environment^42,51^. In this study, we employed the murine strain UMB-MBP-01, which demonstrates an anti-inflammatory and pro-homeostatic effect^33,34^, and porcine-isolated *B. pseudolongum* strain ATCC25526 to investigate the strain-specific mechanisms of host responses in culture and in vivo. The distinct genetic attributes and immunomodulatory capabilities between UMB-MBP-01 and the ATCC25526 show that *B. pseudolongum* modulates intestinal responses and host immunity in a strain-specific manner. Using our clinically relevant murine model, we observed UMB-MBP-01 exerted stronger immunologic effects in intestinal responses mostly likely through regulation of multiple aspects of lymphocyte functions, while ATCC25526 appeared to exert immunomodulatory effects, at least in part, via stimulation of phagocytosis and induced lipid metabolism. We further demonstrated the in culture that *B. pseudolongum* elicited strain-specific activation and cytokine responses in isolated DC and MΦ. *B. pseudolongum* also uniquely changed in local and systemic leukocyte distribution and LN morphology in vivo, demonstrating the unique immune modulatory effects of the two strains. Furthermore, in the small intestine segments, UMB-MBP-01 was most impactful in modulating Tregs, while ATCC25526 most pronouncedly increased DCs. We speculate that these strain-specific immunomodulatory effects are rooted in their niche adaption due to the different mammalian gut microenvironments from which they were derived. This reinforces the importance of understanding strain-specific immunomodulatory properties and host tropism that underline the beneficial effects of probiotics, which is fundamentally critical to inform selection of probiotic strains as therapeutic targets.

It remains unclear whether *B. pseudolongum* immune and intestinal modulation is mediated through direct interactions with the intestinal epithelium, or indirectly via modulation of endogenous gut microbiome with consequent effects on intestinal metabolism and immunity, or both^18^. In our study, the administration of two separate *B. pseudolongum* strains resulted in profoundly different gut microbiomes in both structure and functional capabilities as well as intestinal responses, suggesting the critical involvement of the endogenous gut microbiome as a key element of their immunomodulatory attributes and indicating likely indirect effects. Future investigations on the functional output of the gut microbial community would provide important insights on the mechanistic role of gut microbiome that may critically contribute to host regulation. Our results align with recent key clinical findings, suggesting that *Bifidobacterium* could act as a “microbiome modulator” to competitively exclude toxigenic pathogens and orchestrate homeostatic gut metabolism and host immune responses^52–55^. It is worth noting that the effect of LBPs on the structure and function of the microbial community has not been accepted as a standard parameter for characterizing or evaluating LBP efficacy. Our study emphasizes the importance of understanding the dynamics of the gut microbial community to understand the efficacy and specificity of LBPs. Based on the results in this and our previous study^33^, we speculate that microbiome-driven immunomodulatory effects can be attributed to one or a few keystone bacterial strain(s). This speculation may be critical for future probiotics design and evaluation in both animal and clinical studies. Our data demonstrated the significant impact of a single keystone bacterial strain driving profound local and systemic changes. To thoroughly characterize such probiotic strains, not only is it important to examine both strain-specific genomic features and immunomodulatory properties, but also it is pivotal to understand the gut microbiome of the recipients and gut responses to microbiome changes, that together determine the efficacy of immunomodulation by probiotic strains. This is also an essential prerequisite for probiotics to be adapted clinically to improve pro-tolerogenic effects in peri-, pre- or post-organ transplantation, eventually to improve long-term allograft survival and overall quality of life of transplant recipient.

Distinctive from ATCC25526 and a pro-inflammatory bacterial control, UMB-MBP-01 demonstrated strain persistence within the gut microbiome after administration, further highlighting the role of gut microbiome in LBP-host interaction. Previous studies suggested that the administration of *B. longum* subsp*. longum* had stable, persistent colonization in recipients whose gut microbiome previously had low abundance of gene content involved in carbohydrate utilization, suggesting competition for resources as a key mechanism determining strain persistence^56^. This may relate to the ecological concept of “colonization resistance’, whereby endogenous microbiota occupy host tissues with an intrinsic capability to limit the introduction of exogenous microorganisms and the expansion of endogenous microorganisms, while a microbiota with low capacity in carbohydrate assimilation could be more permissive to exogenous colonization that can fundamentally disrupt the microbiota^57,58^. Given human gut reliance on microbiota to cope with glycan-rich gut environment for the metabolism of luminal oligosaccharides^59^, the ability of *Bifidobacterium* strains to utilize complex carbohydrates provides a selective advantage to effectively compete for nutrients with other bacteria in the gut microenvironment^59^. Interestingly, their repertoire of glycoside hydrolases are species- or strain-specific^50,60^, indicating indispensable roles for glycan-assimilation in their specific niche adaptability to the intestinal microenvironment. Supporting the competitive exclusion ecological theory, we observed the depletion of endogenous gut microorganisms *Muribaculaceae* and *Erysipelotrichaceae* with *Bifidobacterium* treatment, as well as the specialized carbohydrate metabolism of UMB-MBP-01 in utilizing a greater variety of oligosaccharide molecules and host-derived glycans. On the other hand, we observed the increased abundance of *Klebsiella michiganensis* and *Clostridium* in the gut microbiome upon ATCC25526 administration. As *K. michiganensis* and *K. oxytoca* have been demonstrated to cause colonization resistance to multi-drug resistant Enterobacteriaceae gut invasion and *Clostridium scindens* can also mediate resistance to pathogenic strains such as *C. difficile*^61–63^, this result suggests the colonization resistance to other potential pathogenic strains can be one potential protective mechanisms via which ATCC25526 exerts probiotic effects^64^. These data again emphasize the varied, strain-specific mechanisms of probiotic strains. Future longitudinal characterization of strain persistence, microbiota changes, and oligosaccharide and glycoprotein assimilation may hold a key to determine probiotic strain specificity in host adaption and intestinal responses.

The bacterial determinants of immunomodulation properties of individual strains remain an underdeveloped research area. Recent studies revealed strains of the same species induced different immunophenotypes, suggesting that bacteria-induced immunomodulation is not dictated by bacterial phylogeny^22,65^. Multiple LBP produced effector molecules that interact with host immunity have been recently identified^66^. In particular, cell wall components found in Gram-positive bacteria, such as peptidoglycan and lipoteichoic acid, contain MAMPs which are recognized by immunoregulatory pattern recognition receptors such as Toll-like receptors^23^. *Bifidobacterium* peptidoglycans have also demonstrated immunomodulatory effects on the Th1 polarization of naïve T cells as well as DC maturation and enhanced immune responses^67,68^. Other surface molecules such as EPS and pili also play a role in *Bifidobacterium’s* strain-specific pro-homeostatic immunomodulation^27,28^. We also observed here expanded enzymatic capabilities of UMB-MBP-01, but not ATCC25526, in assimilating oligosaccharide molecules and host-derived glycans as well as the capacity to manufacture cell wall components such as peptidoglycan. Together these studies implicate the genetic variation of different bacterial strains as underlying induced intestinal responses and mucosal immunological signaling. As the cell wall components were generally considered a key bacterial determinant in *Bifidobacterium* immune modulation capabilities, we speculate other important determinants may also play a crucial role, for instance, the specific bioactive metabolites and pathways through which the bacterial strains exert their immunomodulatory effects, and the specific intestinal cell types that respond to those signals. This speculation warrants future experimental validation and mechanistic determination for this underdeveloped research area.

Making conclusions about the immunomodulatory effects of bacterial strains based on the surface structures alone is inaccurate, as the preparations of the cell wall components and extracellular polysaccharides are strongly influenced by cultivation conditions^69^. The differences observed in this study between *Bifidobacterium* and purified EPS in culture versus in vivo indeed suggest the presence of many other molecules and mechanisms that contribute to the regulation of immunity and inflammation. In particular, the observation that EPS alone yielded gut Treg recruitment in addition to innate myeloid cell populations suggests that EPS may act indirectly through an intermediary, such as intestinal epithelial cells, which then influence leukocyte subsets. Indeed, characterization of *B*. *pseudolongum*-induced innate immune responses revealed that these bacteria induce a more balanced anti-inflammatory and homeostatic cytokine response from DC and MΦ^33^. In addition, *B. pseudolongum* induced changes in LN architecture, resulting in an increased ratio of extracellular matrix protein laminin α4 to laminin α5 in the CR, a microdomain structure that is mechanistically associated with immunologic suppression and tolerance^33^. These observations demonstrate that a single probiotic bacterial strain can influence local, regional, and systemic immunity through both innate and adaptive pathways. A holistic understanding of the strain-specific bacterial effects is critical to inform probiotic design as well as immunomodulatory therapeutic targeting.

## Conclusion

The distinct genetic attributes and immunomodulatory capabilities of UMB-MBP-01 compared to *B. pseudolongum* type strain ATCC25526 show that *Bifidobacterium* modulates intestinal responses and host immunity in a strain-specific manner. Our results highlight the importance to characterize individual *Bifidobacterium* strains and not to generalize their immunomodulatory effects to other strains of the same species, despite their many shared features. It is critical to investigate both endogenous microbiota in response to LBP strains and to profile the intestinal responses, in order to interrogate mechanistically the highly coordinated multicellular host-microbe interactions, which are key to understanding strain-specific immunomodulation. Future studies are warranted to investigate the specific bioactive metabolites and pathways through which the gut microbiota exert their immunomodulatory effects, and the specific intestinal cell types that respond to those signals. A comprehensive understanding of strain-specific immunomodulatory properties is fundamentally important to inform probiotic design as well as immunomodulatory therapeutic targeting.

## Methods and materials

### Strains cultivation and genomic sequencing

*Bifidobacterium pseudolongum* strain ATCC25526 was purchased from ATCC (Manassas, Virginia). *B. pseudolongum* strain UMB-MBP-01 was isolated from the feces of C57BL/6 J mice through passages and screening on Bifidus Selective Medium (BSM) agar (Sigma-Aldrich, St. Louis, MO, USA), as previously described^34^. Both strains were initially grown anaerobically at 37 °C for 3–5 days on Bifido Selective Media (BSM) agar plates (Millipore Sigma, Burlington, MA), from which a single colony was selected and grown in BSM broth (Millipore Sigma, 90,273-500G-F) until stationary phase (up to 3 days). *Desulfovibrio desulfuricans subsp. desulfuricans* (ATCC27774) was purchased from ATCC and grown in ATCC Medium: 1249 Modified Baar’s Medium (MBM) for sulfate reducers, which was made according to ATCC protocol. Cultures were initially incubated under anaerobic conditions for 5 days on Modified Baar’s Medium agar plates, after which single colonies were chosen, transferred to liquid media, and incubated for up to 3 weeks. *B. pseudolongum* strains ATCC25526 and UMB-MBP-01 were used in cell stimulation and cytokine assays. To ensure the integrity of the cultivated strain and to screen for potential contamination, each batch of bacterial cultivation was examined carefully using gram stain to check the morphotype and periodically using genome sequencing. A genomic similarity score lower than 99.9% to the original culture’s genome sequence would raise a flag for further investigation and quality check.

*Bifidobacterium pseudolongum* UMB-MBP-01 was sequenced previously^34^. For strain ATCC25526, genomic DNA extraction was performed using a lysozyme/mutanolysin-based cell lysis followed by purification using the Wizard Genomic DNA Purification Kit (Promega, Madison, WI, USA). Library preparation on extracted DNA was conducted using a Kapa kit (Roche, Indianapolis, IN) for 150-bp paired-end sequencing, and sequencing was performed with an Illumina (San Diego, CA) MiSeq system. Sequencing was performed by the University of Maryland School of Medicine, Institute for Genome Sciences, Genomics Resource Center with standard operating procedures and assembled using SPAdes v3.14.0^70^. Contig ordering was performed using MAUVE contig mover^71^ and the UMB-MBP-01 genome as reference^72^.

### Exopolysaccharide (EPS) Isolation

EPS was extracted from strains X and Y using the protocol previously published by Bajpai and colleagues^73^. Briefly, bacterial cultures were grown in 500 ml BSM media to early stationary phase, after which trichloroacetic acid was added (14% v/v final) and the mixture incubated at 37 °C for 40 min. After centrifugation at 8,000 g for 20 min at 4 °C, the supernatant was collected, absolute ethanol added (2:1 v/v EtOH:sup), followed by incubation at 4 °C for 48 h and centrifugation at 8,000xg at 4 °C for 20 min. This ethanol wash step was repeated to remove any impurities, and a final centrifugation at 8,000xg at 4 °C for 20 min was performed. The resulting pellet was then dissolved in 5 to 10 ml of water, before being dialyzed against DI water for 48 h, and lyophilized.

### Anaerobic microplate assay

Anaerobic microplates (AN plates, Biolog, Hayward, CA) pre-coated with 95 various carbon sources was used in the assay. Each well of the 96-well AN Biolog plate was coated by a sole-carbon source, with one well being used as no carbon control. Metabolism of the substrate in particular wells results in formazan production, producing a color change in the tetrazolium dye. Cultured bacteria in log growth phase were centrifuged down to pellet, which was suspended using inoculating fluid (Biolog, Hayward, CA) to an OD value around 0.26. The suspension was added to the microplates and sealed by anaerobic GasPak EZ anaerobe gas pouch system with anaerobic indicators (BD, Franklin Lakes, NJ). The plates were read between 20 and 24 h following inoculation with a pre-grown isolate using spectrophotometer microplate reader (Molecular Devices, LLC, San Jose, CA) and reading data acquisition was performed using SoftMax Pro 7 software version 7.1.0 build 246,936 (Molecular Devices, LLC, San Jose, CA). The procedure was performed in triplicate for each strain. The no carbon control reading was subtracted from each of the readings of the wells, and student’s t test was performed to test if the average reading was significantly different from zero.

### Comparative genomics analyses

A total of 79 *B. pseudolongum* genomes were included in the analyses, which included the four sequenced in this study and all 75 available *B. pseudolongum* genomes on GenBank (retrieved September 2021, Supplemental Table 5A). The pangenome was constructed using anvi’o vers 6.2 workflow, an open-source analysis and visualization platform for microbial 'omics^74,75^. Briefly, this workflow (1) dereplicates genomes based on similarity score calculated using Sourmash vers 3.3^76^, (2) uses BLASTP to compute ANI identity between all pairs of genes, (3) uses the Markov Cluster Algorithm (MCL)^77^ to generate homologous gene clusters (HGCs) based on all-versus-all sequence similarity, and (4) aligns amino acid sequences using MUSCLE^78^ for each gene cluster. Each gene was assigned to core or accessory according to the hierarchical clustering of the gene clusters. Sourmash vers 3.3^76^ was used to compute ANI across genomes. Functional annotation of each secreted protein was performed employing the eggNOG database v5.0^79^ using eggNOG-mapper v2^80^ and the results were imported into the anvi’o contig database. Further functional annotation included PFAMs based on hidden Markov model (HMM) search to Pfam vers34.0^81^. Protein-coding genes were also annotated to metabolic functionality categories using KEGG (Kyoto Encyclopedia of Genes and Genomes)^82^. GhostKOALA annotation tool^83^ was used to assign KEGG Identifiers. Enrichment analyses were performed using Anvi’o pangenome pipeline that take COG functions across genomes and clade affiliation as the explanatory variable. The equality of proportions across clade affiliation was tested using a Rao score test, which generates an enrichment score as the test statistic and a p value. The q-value was then calculated from the p value to account for multiple testing using R package q-value^84^. A COG function was considered enriched if the q-value was below 0.05.

The prediction of genes encoding extracellular enzymes possessing structurally related catalytic and carbohydrate-binding modules catalyzing hydrolysis, modification, or synthesis of glycoside bounds was performed using dbCAN2^85^ and dbCAN HMMdb (v.9) that was built using CAZy database (v.07302020)^49^. To identify signal-peptide specific sequence motifs, we employed the subcellular localization prediction tool PSORTb (v.3.0.2)^86^.

### Cell culture

Peritoneal macrophages (MΦ) and bone marrow derived dendritic cells (BMDCs) were isolated from C57BL/6 mice and then seeded onto 24 well plates in 1 ml RPMI complete medium as described^87^. Briefly, MΦ were collected 4 days after i.p. injection of Remel Thioglycollate solution (Thermo Fisher Scientific, Waltham, MA). BMDCs were generated from bone marrow cells treated with 10 ng/ml GM-CSF (R&D Systems, Minneapolis, MN) for 10 days. Loosely adherent immature BMDCs were collected and then CD11c + DCs were enriched using CD11c positive selection kit (Stemcell Technologies, Cambridge, MA). Twenty-four hours after culture of purified subsets, the cells were stimulated with UV-killed *Bifidobacterium* bacteria or purified EPS for 24 h. Bacterial cells were killed by UV exposure at 100 μJ/cm2 for four 15-min cycles with a UV CrossLinker (Fisher Scientific, Hampton, NH). Culture supernatants were collected from whole bacteria or EPS stimulated cultures and ELISA for TNFα, IL-6, and IL-10 (BioLegend, San Diego, CA) performed. Myeloid cells from co-culture wells were collected and analyzed by flow cytometry. Costimulatory molecule flow cytometry analysis for MΦ and BMDC used singlet^+^F4/80^+^ and singlet^+^CD11c^+^ populations, respectively.

### Flow cytometry

Cells were passed through 70-μm nylon mesh screens (Thermo Fisher Scientific, Waltham, MA) to produce single-cell suspensions. Cell suspensions were treated with anti-CD16/32 (clone 93, eBioscience) to block Fc receptors, and then stained for 30 min at 4 °C with antibodies against surface molecules (Supplementary Table 9) and washed 2 times in FACS buffer [phosphate buffered saline (PBS) with 0.5% w/v Bovine serum albumin (BSA). Samples were analyzed with an LSR Fortessa Cell Analyzer (BD Biosciences), and data analyzed with FlowJo software version 10.6 (BD Biosciences).

### Immunohistochemistry

Peripheral LN, mesenteric LN, and small bowel segment (duodenal-jejunal junction) were excised and washed in cold PBS before freezing in OCT (Sakura Finetek, Torrance, CA) in histology blocks on dry ice and then stored at -80 °C. LN cryosections were cut in triplicate at 5 μm using a Microm HM 550 cryostat (Thermo Fisher Scientific, Waltham, MA). Sections attached to slides were fixed with cold acetone/ethanol (1:1) solution and washed in PBS buffer (Lonza, Morristown, NJ). Primary antibodies and isotype controls (Supplementary Table 9) were added to slides for 1 h in a humidified chamber. Sections were washed with PBS, blocked with 2.5% donkey serum and 2.5% goat serum, and incubated with secondary antibodies for 60 min. Slides were then fixed with 4% paraformaldehyde/PBS (Alfa Aesar, Haverhill, MA) for 5 min, incubated with 1% glycerol for 5 min, and Prolong Gold Antifade Mountant with or without DAPI (Thermo Fisher Scientific, Waltham, MA) was added before applying cover slips. Images were acquired using an Accu-Scope EXC-500 fluorescent microscope (Nikon, Melville, NY) and analyzed with Volocity image analysis software (PerkinElmer, Waltham, MA). Mean fluorescence intensity (MFI) was calculated within demarcated high endothelial venules (HEV) and cortical ridge (CR) regions of mLN and pLN as well as of whole intestinal images. MFI quantified based on at least 2 independent experiments with 3 mice/group, 3 LNs/mouse or 2 intestine segments/mouse, 3 sections/LN or intestinal segments, and 3–5 fields/section, i.e. at least 20 total microscopy fields per condition over 2 experiments.

### Mice experiments

Female C57BL/6 mice between 8 and 14 weeks of age were purchased from The Jackson Laboratory (Bar Harbor, ME). All the procedures involving mice were performed in accordance with the guidelines and regulations set by the Office of Animal Welfare Assurance of the University of Maryland School of Medicine (UMSOM), under the approved IACUC (institutional animal care and use committee) protocols 0,518,004 and 0,121,001. Mice were fed antibiotics (kanamycin, gentamicin, colistin, metronidazole, and vancomycin) ad libitum in drinking water on days −6 to −1. On day 0, cultured *Bifidobacterium* ATCC25526 or UMB-MBP-01 were gavaged orally. Mice received tacrolimus (3 mg/kg/d subcutaneously) on days 0 and 1. On day 2, the animals were euthanized by CO_2_ narcosis. Mesenteric and peripheral (axillary, inguinal, popliteal, brachial) LNs as well as small intestine were harvested. Fecal pellets were also collected prior to euthanasia into individual tubes, using aseptic technique to minimize handling, and stored at −80 °C. Antibiotics were USP grade or pharmaceutical secondary standard (all from MilliporeSigma): kanamycin sulfate (0.4 mg/ml), gentamicin sulfate (0.035 mg/ml), colistin sulfate (850 U/ml), metronidazole (0.215 mg/ml), and vancomycin hydrochloride (0.045 mg/ml) were dissolved in vivarium drinking water and administered ad libitum. Tacrolimus (USP grade, MilliporeSigma) was reconstituted in DMSO (USP grade, MilliporeSigma) at 20 mg/ml and diluted with absolute ethanol (USP grade, Decon Labs, King of Prussia, PA) to 1.5 mg/ml. DMSO/ethanol stock was diluted 1:5 in sterile PBS for s.c. injection and injected at 10 μl/g (3 mg/kg/day). Mouse experiments are described according to ARRIVE guidelines (https://arriveguidelines.org).

### Metagenomic sequencing and microbiome analyses

Luminal fecal contents were harvested from fresh intestine and stored immediately in DNA/RNA Shields (Zymo Research, Irvine, CA), proved to be a reliable solution for stool sample preservation to stabilize and protect the integrity of nucleic acids^88,89^. The colon content from ~ 1 cm colon tissue was used in DNA extraction. Metagenomic sequencing libraries were constructed from the same DNA using the Nextera XT Flex kit (Illumina) according to the manufacturer recommendations. Libraries were then pooled together in equimolar proportions and sequenced on a single Illumina NovaSeq 6000 S2 flow cell at the Genomic Resource Center of the Institute for Genome Sciences at the University of Maryland School of Medicine.

Metagenomic sequence reads were removed using BMTagger v3.101^90^ using a Genome Reference Consortium Mouse Build 39 of strain C57BL/6 J (GRCm39)^91^. Sequence read pairs were removed even if only one of the reads matched to the mice genome reference. The Illumina adapter was trimmed and quality assessment was performed using default parameters in fastp (v.0.21.0)^92^. The taxonomic composition of the microbiomes was established using Kraken2 (v.2020.12)^93^ and Braken (v. 2.5.0)^94^ using the comprehensive mouse gut metagenome catalog (CMGM)^40^ to calculate the metagenomic taxonomic composition. The Phyloseq (v.1.34.0)^95^ R package was used to generate the diversity plot and barplot. Linear discriminant analysis (LDA) effect size (LEfSe) analysis^41^ was used to identify fecal phylotypes that could explain differences between. For LEfSe, only taxonomic groups present in > 1% of at least one sample were included in the analyses; the alpha value for the non-parametric factorial Kruskal–Wallis (KW) sum-rank test was set at 0.05 and the threshold for the logarithmic LDA model^96^ score for discriminative features was set at 2.0. An all-against-all BLAST search in multi-class analysis was performed. Metagenomics dataset was mapped to the protein database UniRef90^97^ to ensure the comprehensiveness in functional annotation, and was then summarized using HUMAnN2 (Human Microbiome Project Unified Metabolic Analysis Network) (v0.11.2)^98^ to efficiently and accurately determine the presence, absence, and abundance of metabolic pathways in a microbial community. Further, HUMAnN2 employed a tiered search strategy enabling a species-resolved functional profiling of metagenomes, hence to characterize the contribution to the functional pathways of a known species. Canonical Correspondence Analysis (CCA) was used in ordination analysis, and biplot was generated using vegan package^99,100^ based on bray–curtis distance. CA1 and CA2 were selected as the major components based on the eigenvalue. A species score was scaled proportional to the eigenvalues representing the direction from the origin where the group had a larger than average value for the particular species^99,101^. The species scores greater than 1 were used to select the species that were considered the most significant contributors to each group.

### RNA isolation, transcriptome sequencing and analyses of the intestinal tissues

Dissected intestinal tissues were stored immediately in RNAlater solution (QIAGEN) to stabilize and protect the integrity of RNA^102^. Specimens were stored at −80 °C until extraction. For each sample, bulk RNA was extracted from ~ 1 cm of intestine tissues. Prior to the extraction, 500 µl of ice-cold RNase free PBS was added to the sample. To remove the RNAlater, the mixture was centrifuged at 8,000x*g* for 10 min and the resulting pellet resuspended in 500 µl ice-cold RNase-free PBS with 10 µl of β-mercaptoethanol. Tissue suspension was obtained by bead beating procedure using the FastPrep lysing matrix B protocol (MP Biomedicals, Solon, OH) to homogenized tissues. RNA was extracted from the resulting suspension using TRIzol Reagent (Invitrogen, Carlsbad, CA) following the manufacturer recommendations and followed by protein cleanup using Phasemaker tubes (Invitrogen) and precipitation of total nucleic acids using isopropanol. RNA was resuspended in DEPC-treated DNAase/RNAase-free water. Residual DNA was purged from total RNA extract by treating once with TURBO DNase (Ambion, Austin, TX, Cat. No. AM1907) according to the manufacturer’s protocol. DNA removal was confirmed via PCR assay using 16S rRNA primer 27 F (5′-AGAGTTTGATCCTGGCTCAG-3′) and 534 R (5′-CATTACCGCGGCTGCTGG-3′). The quality of extracted RNA was verified using the Agilent 2100 Expert Bioanalyzer using the RNA 1000 Nano kit (Agilent Technologies, Santa Clara, CA). Ribosomal RNA depletion and library construction were performed using the RiboZero Plus kit and TruSeq stranded mRNA library preparation kit (Illumina) according to the manufacturer’s recommendations. Libraries were then pooled together in equimolar proportions and sequenced on a single Illumina NovaSeq 6000 S2 flow cell at the Genomic Resource Center (Institute for Genome Sciences, University of Maryland School of Medicine) using the 150 bp paired-end protocol.

Bioinformatic analysis of the transcriptome data includes the quality of fastq files, which was evaluated by FastQC. Reads were aligned to the mouse genome (v. Mus_musculus.GRCm39) using HiSat (v. HISAT2-2.1.0)^103^ and the number of reads that aligned to the coding regions were determined using HTSeq (v.1.0.0)^104^. Significant differential expression was assessed using DEseq with an FDR value ≤ 0.05^105^. Quadrant plot to show whether DEGs have the same or opposite relationships between each of the pairwise comparisons of UMB-MBP-01 vs ATCC25526 and UMB-MBP-01 vs control. Gene Ontology (GO) enrichment analysis was performed in order to identify GO terms significantly over-represented in genes deregulated in specific comparisons and, as a result, to suggest possible functional characteristics of these genes. Enriched GO terms in the set of genes that are significantly over-expressed or under-expressed in a specific condition may suggest possible mechanisms of regulation or functional pathways that are, respectively, activated or repressed in that condition. Over-representation analyses^106^ of differentially expressed genes (DEGs) against GO ontologies was performed using enrichGO function of clusterProfile Bioconductor package^107^. Cnetplot function was used to depict the linkages of genes and GO terms as a Gene-Concept Network for top over-represented GO terms based on q-value and gene-count.

### Ethical approval and consent to participate

All animal studies were carried out in accordance with IACUC-approved protocols.

## Supplementary Information

Supplementary Information 1.

Supplementary Information 2.

Supplementary Information 3.

Supplementary Information 4.

Supplementary Information 5.

Supplementary Information 6.

Supplementary Information 7.

Supplementary Information 8.

Supplementary Information 9.

Supplementary Information 10.

Supplementary Information 11.

## Data Availability

The assembly of the genomes (accession numbers were listed in Supplemental Table 2), metagenome (SRP361281), and transcriptome sequences were submitted to GenBank under BioProject PRJNA809764 (https://www.ncbi.nlm.nih.gov/bioproject/PRJNA809764). R codes and input dataset used to generate the heatmap was deposited in github (https://github.com/igsbma/genome_paper). Immunologic experimental raw data files available to download, the DOIs links for Fig. 1–3 and Supplemental 4 were in their corresponding figure legend.
