# Author Correction: Strain-specific alterations in gut microbiome and host immune responses elicited by tolerogenic *Bifidobacterium pseudolongum*

**DOI:** 10.1038/s41598-023-33772-1

**Published:** 2023-04-24

**Authors:** Bing Ma, Samuel J. Gavzy, Vikas Saxena, Yang Song, Wenji Piao, Hnin Wai Lwin, Ram Lakhan, Jegan Iyyathurai, Lushen Li, Michael France, Christina Paluskievicz, Marina W. Shirkey, Lauren Hittle, Arshi Munawwar, Emmanuel F. Mongodin, Jonathan S. Bromberg

**Affiliations:** 1 Institute of Genome Sciences, University of Maryland School of Medicine, Baltimore, MD 21201 USA; 2 Department of Microbiology and Immunology, University of Maryland School of Medicine, Baltimore, MD 21201 USA; 3 Department of Surgery, University of Maryland Medical Center, Baltimore, MD 21201 USA; 4 Center for Vascular and Inflammatory Diseases, University of Maryland School of Medicine, Baltimore, MD 21201 USA; 5 Institute of Human Virology, University of Maryland School of Medicine, Baltimore, MD 21201 USA; 6 Present Address: Division of Lung Diseases, National Heart, Lung, and Blood Institute (NHLBI), National Institutes of Health (NIH), Bethesda, MD USA

Correction to: *Scientific Reports* 10.1038/s41598-023-27706-0, published online 19 January 2023

The original version of this Article contained an error in the spelling of the author Jegan Iyyathurai which was incorrectly given as Jegan Lyyathurai.

The original Article has been corrected.

