# Supplementary material for: Strain-specific alterations in gut microbiome and host immune responses elicited by tolerogenic Bifidobacterium pseudolongum
Source: Sci Rep. 2023 Jan 19;13:1023. doi: 10.1038/s41598-023-27706-0 (PMC9852428; doi:10.1038/s41598-023-27706-0)
Supplement: Supplementary file 1 — Supplementary Information 1. [file 41598_2023_27706_MOESM1_ESM.pdf]

A. UMB-MBP-01 vs. control up-regulated

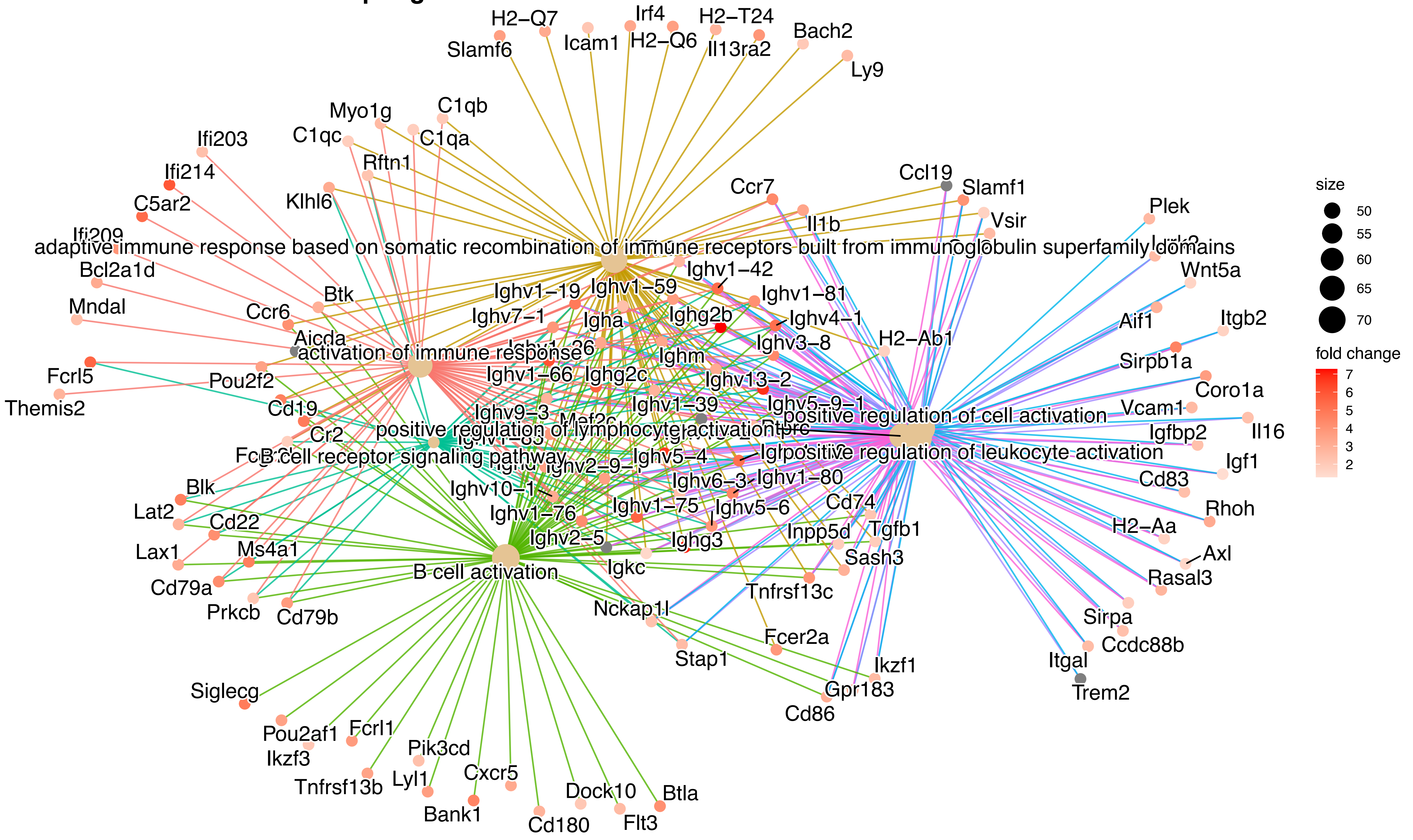

B. UMB-MBP-01 vs. control down-regulated

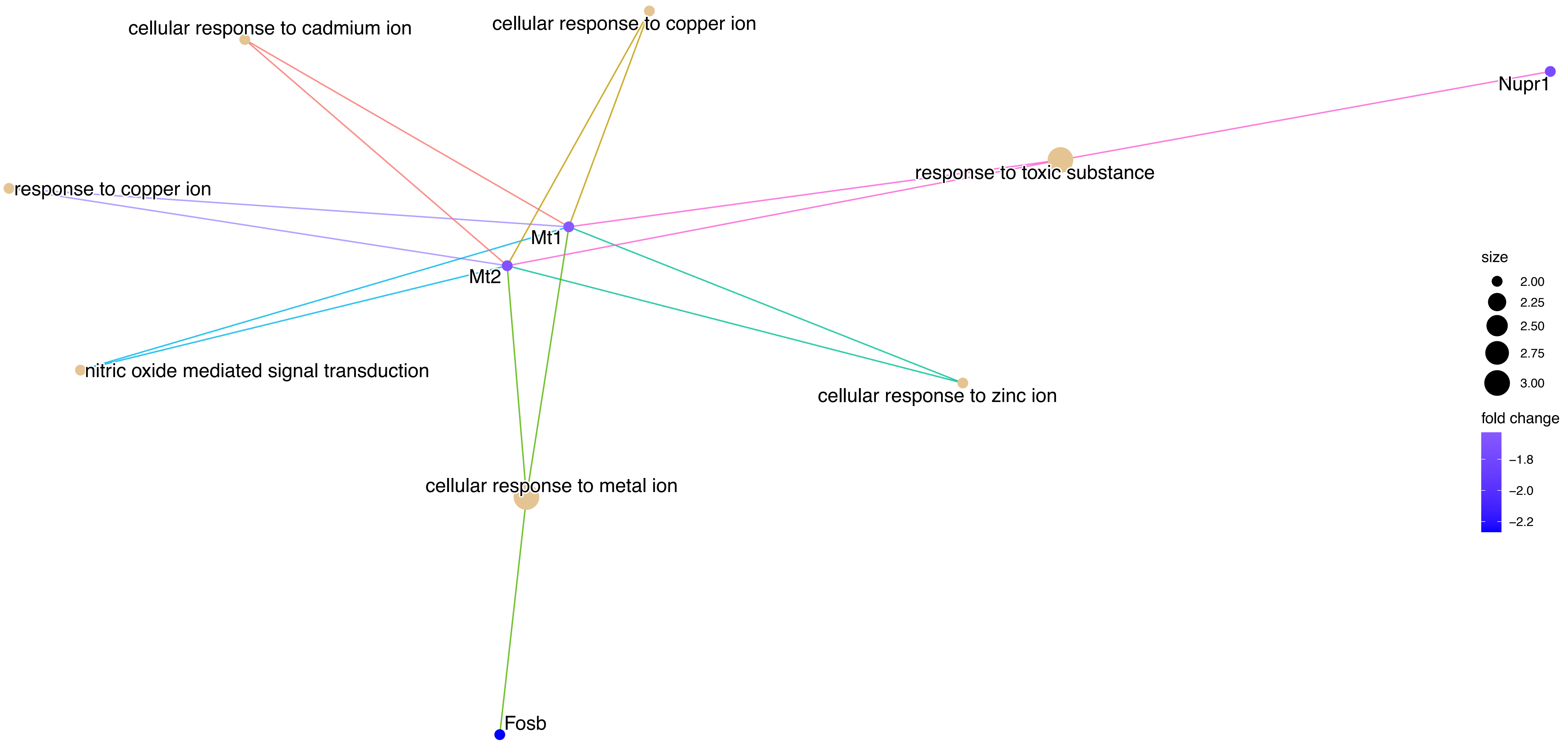

C. UMB-MBP-01 vs. ATCC25526 up-regulated

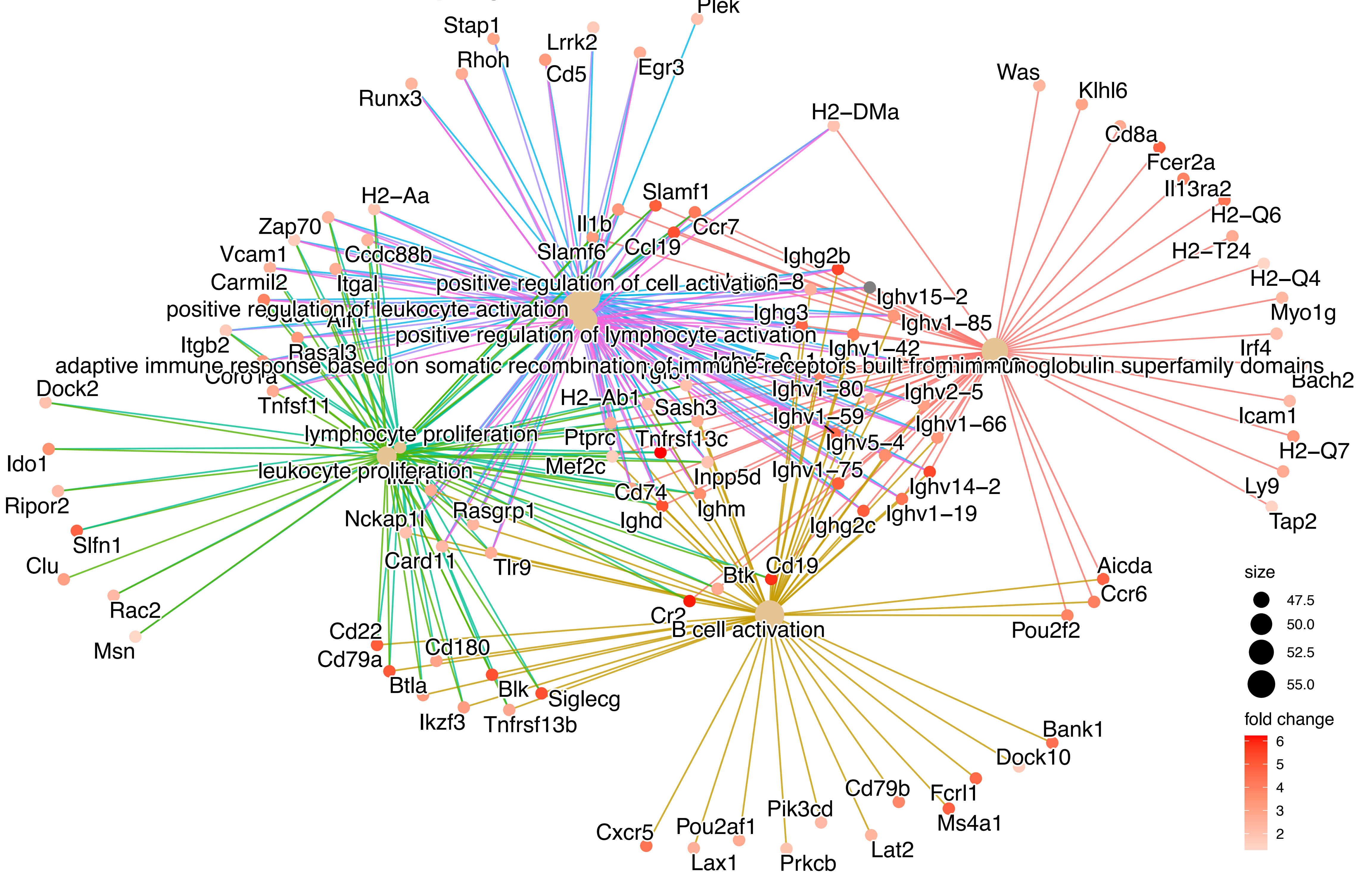

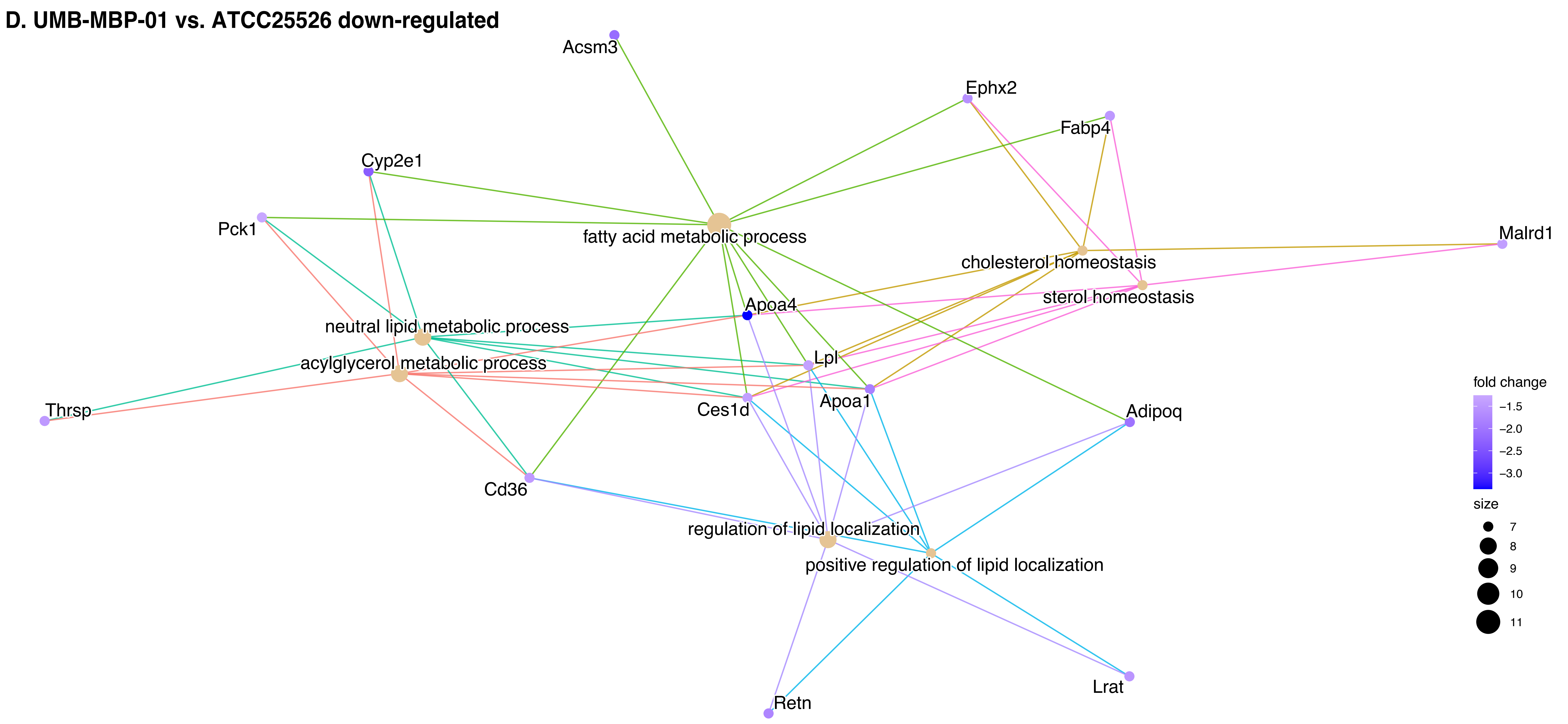
D. UMB-MBP-01 vs. ATCC25526 down-regulated
Acsm3
Ephx2
Fabp4
Cyp2e1
Pck1
fatty acid metabolic process
Malrd1
cholesterol homeostasis
sterol homeostasis
Apoa4
neutral lipid metabolic process
Lpl
acylglycerol metabolic process
Apoa1
Ces1d
Thrsp
Adipoq
Cd36
regulation of lipid localization
positive regulation of lipid localization
Lrat
Retn
fold change
−1.5
−2.0
−2.5
−3.0
size
7
8
9
10
11

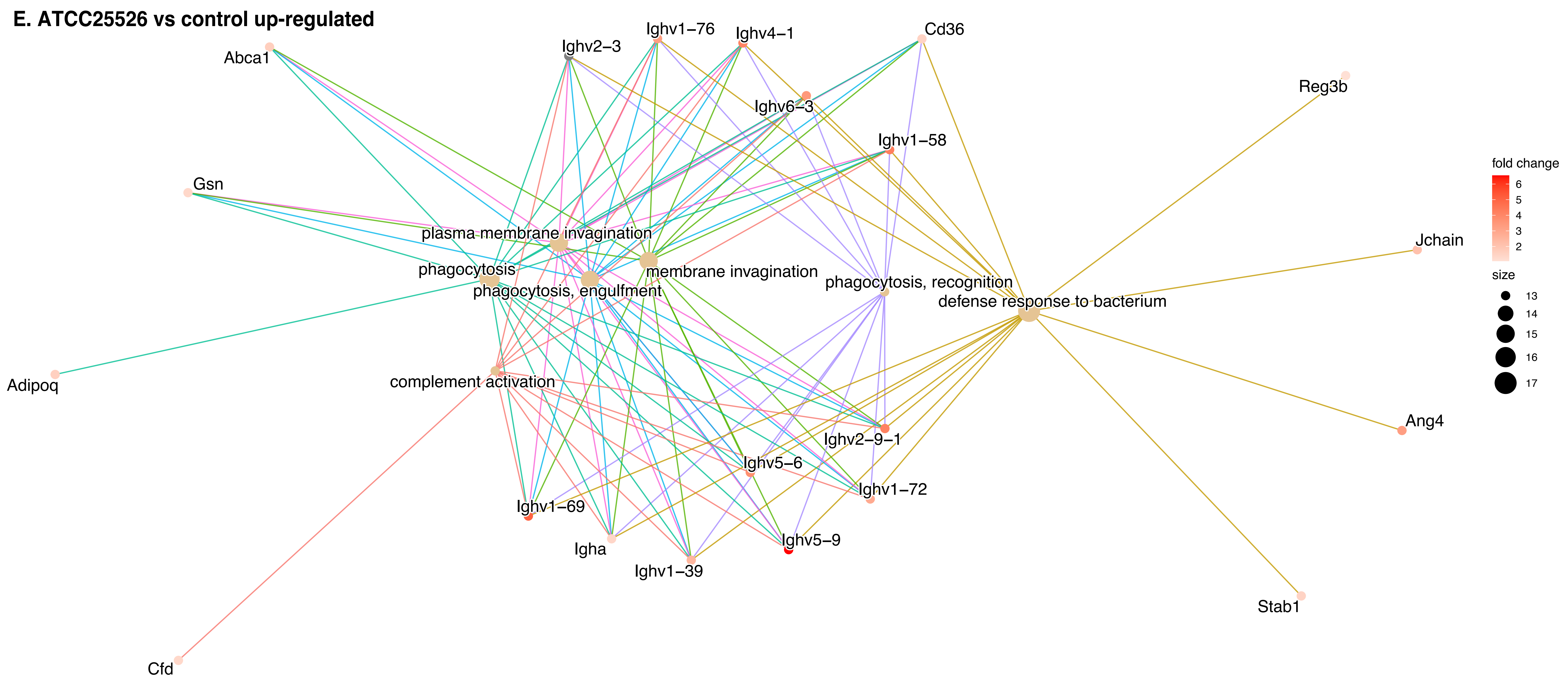
E. ATCC25526 vs control up-regulated
Abca1
Ighv2−3
Ighv1−76
Ighv4−1
Cd36
Reg3b
Ighv6−3
Ighv1−58
Gsn
plasma membrane invagination
phagocytosis
membrane invagination
phagocytosis, engulfment
phagocytosis, recognition
defense response to bacterium
Jchain
Adipoq
complement activation
Ighv2−9−1
Ang4
Ighv5−6
Ighv1−72
Ighv1−69
Igha
Ighv5−9
Ighv1−39
Stab1
Cfd
fold change
6
5
4
3
2
size
13
14
15
16
17

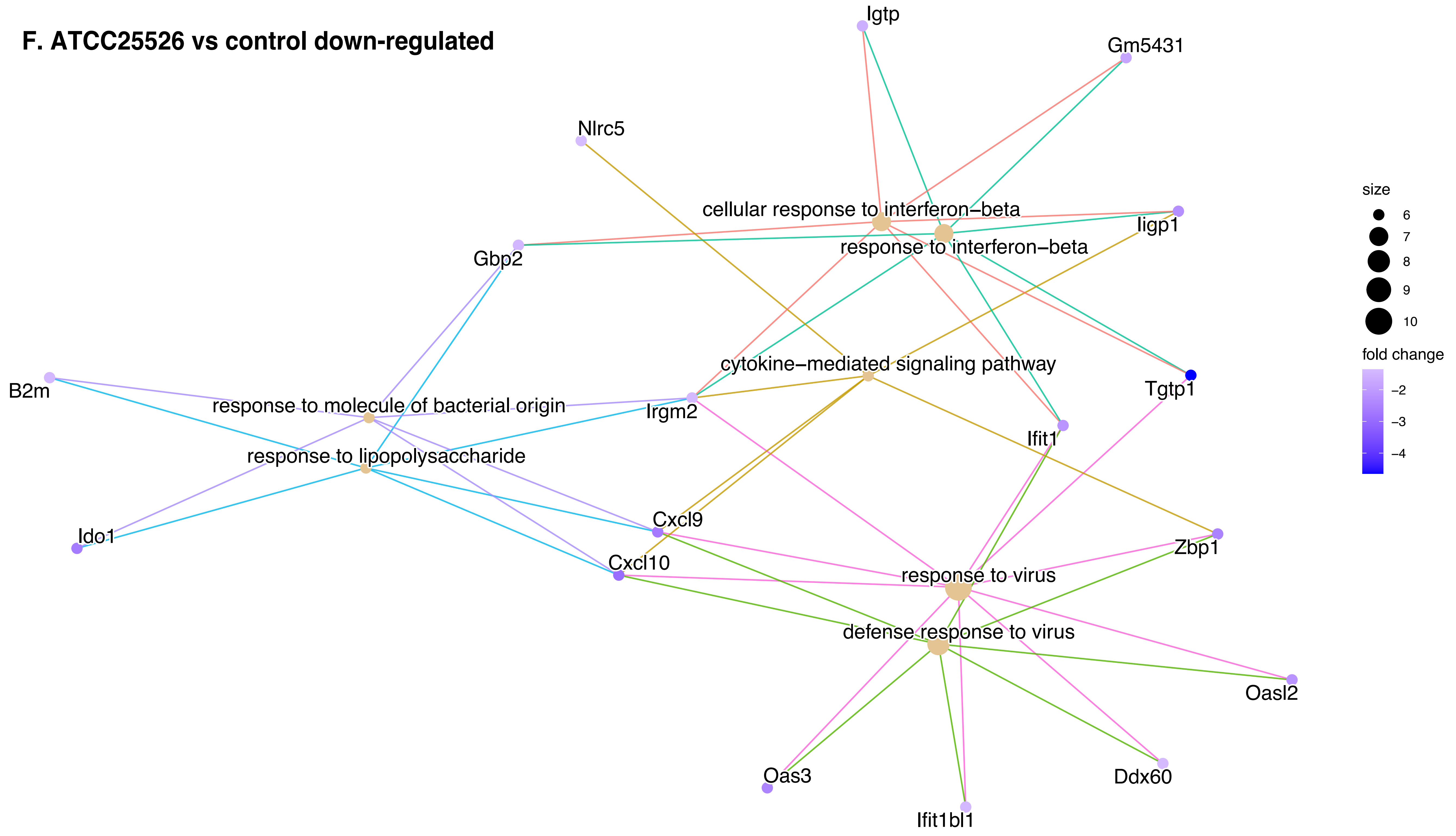
F. ATCC25526 vs control down-regulated
Igtp
Gm5431
Nlrc5
cellular response to interferon−beta
response to interferon−beta
Iigp1
Gbp2
B2m
cytokine−mediated signaling pathway
Tgtp1
response to molecule of bacterial origin
Irgm2
Ifit1
response to lipopolysaccharide
Cxcl9
Ido1
Cxcl10
Zbp1
response to virus
defense response to virus
Oasl2
Oas3
Ddx60
Ifit1bl1
size
6
7
8
9
10
fold change
−2
−3
−4
