# Supplementary material for: Strain-specific alterations in gut microbiome and host immune responses elicited by tolerogenic Bifidobacterium pseudolongum
Source: Sci Rep. 2023 Jan 19;13:1023. doi: 10.1038/s41598-023-27706-0 (PMC9852428; doi:10.1038/s41598-023-27706-0)
Supplement: Supplementary file 10 — Supplementary Information 10. [file 41598_2023_27706_MOESM10_ESM.docx]

**Supplemental Table 9: List of primary and secondary antibodies used in this study**

| **Target Molecule** | **Clone** | **Catalog No.** |
| --- | --- | --- |
| ERTR7 | Polyclonal | Santa Cruz; SC-73355 |
| B220 | RA3-6B2 | eBioSc; 17-0452-82 |
| CD4 | GK1.5 | Biolegend; 100401 |
| CD8 | 53-6.7 | Biolegend; 100701 |
| Laminin α4 | 775830 | R&D; MAB3837 |
| Laminin α5 | Polyclonal | Novus Biol; NBP1-18714 |
| CD11c | HL3 | BD; 550283; 553801 |
| CD11b | M1/70 | eBioSc; 11-0112-81 |
| F4/80 | BM8 | eBioSc; 11-4801-81 |
| CD40 | 3/23 | Biolegend; 124609 |
| CD80 | 16-10A1 | Biolegend; 104705 |
| CD86 | GL-1 | Biolegend; 105011 |
| MHC II (I-A/I-E) | M5/114.15.2 | Biolegend; 107619 |
| Foxp3 | PCH101 | eBioSc; 14-4776-82 |
| anti-rabbit IgG AF488 | Polyclonal | Jackson ImmunoResearch; 711-545-152 |
| anti-rabbit DL405 | Polyclonal | Jackson ImmunoResearch; 711-476-152 |
| anti-rabbit IgG AF647 | Polyclonal | Jackson ImmunoResearch; 711-606-152 |
| anti-mouse IgG AF647 | Polyclonal | Jackson ImmunoResearchl 715-605-151 |
| anti-rat IgG AF594 | Polyclonal | Jackson ImmunoResearch; 112-586-143 |
| anti-rabbit AF488 | Polyclonal | Jackson ImmunoResearch; 111-545-003 |
| anti-rabbit IgG AF594 | Polyclonal | Jackson ImmunoResearch; 111-585-003 |
