# Supplementary material for: Strain-specific alterations in gut microbiome and host immune responses elicited by tolerogenic Bifidobacterium pseudolongum
Source: Sci Rep. 2023 Jan 19;13:1023. doi: 10.1038/s41598-023-27706-0 (PMC9852428; doi:10.1038/s41598-023-27706-0)
Supplement: Supplementary file 11 — Supplementary Information 11. [file 41598_2023_27706_MOESM11_ESM.docx]

**Supplemental Figures**

**Supplemental Figure 1.** Proportion of the pangenome categories of *Bifidobacterium* species: *B. breve*, *B. longum* and *B. pseudolongum*. Homologous gene clusters (HGCs) identified based on all-versus-all sequence similarity. Each gene was assigned to core or accessory according to the hierarchical clustering of the gene clusters.

**Supplemental Figure 2.** Glycosyl hydrolases similarity among *B. pseudolongum* genomes. BLAST Score Ratio (BSR) approach [1] with default settings was used to calculate the GH similarity. UMB-MBP-01 was used as the reference to compare to glycosyl hydrolyse capabilities of the other 61 *B. pseudolongum* strains. Color indicates BSR score scaled from 0 (absent) to 100 (identical proteins).

**Supplemental Figure 3.** Flow cytometry gating strategy.

**Supplemental Figure 4.** C57BL/6 mice treated with antibiotics for 6 days and then gavaged with PBS, ATCC25526 (ATCC) or UMB-MBP-01 (MD) *Bifidobacteria*. Mice then treated with tacrolimus (3 mg/kg/d s.c.) for the next two days. Tissues harvested 2 days after bacterial gavage and analyzed by flow cytometry. MLN stained for **A**) B220+ B cells, **B**) CD4+ T cells, **C**) CD8+ T cells, **D**) CD4+Foxp3+ Treg, **E**) CD11c+ DC, and **F**) F4/80+ MΦ. PLN stained for **G**) B220+ B cells, **H**) CD4+ T cells, **I**) CD8+ T cells, **J**) CD4+Foxp3+ Treg, **K**) CD11c+ DC, and **L**) F4/80+ MΦ. Frozen tissue sections analyzed by immunohistochemistry for **M**) CD11c+ DC and **N**) F4/80+ MΦ. **O**) MLN stained for laminin α4 and α5, with their ratio depicted. Treatment groups compared using one-way ANOVA. ** p value < 0.01. Data representative of 2 separate experiments, 3 mice per group, i.e. 6 total mice per condition over 2 experiments. The raw files from these experiments are available to download at <https://doi.org/10.6084/m9.figshare.21685865>.

**Supplemental Figure 5.** Gene-Concept network for most over-represented Gene Ontology (GO) terms depict over-represented functions based on q-value and gene-count. Over-representation analyses [2] of differential expressed genes (DEGs) that are only differentially abundant in **A**) UMB-MBP-001 vs control up-regulated; **B**) UMB-MBP-001 vs control down-regulated; **C**) UMB-MBP-001 vs ATCC25526 up-regulated; **D**) UMB-MBP-001 vs ATCC25526 down-regulated; **E**) ATCC25526 vs control up-regulated; **F**) ATCC25526 vs control down-regulated, using GO ontologies performed using enrichGO function of clusterProfile Bioconductor package[3]. DEGs were determined using log2 fold change (LFC) >(+/-)1 and false discovery rate (FDR)<0.05.

**Supplemental Figure 6.** Transcriptome profiling of intestinal tissues in response to live *Bifidobacterium* cells or *Bifidobacterium* exopolysaccharides (EPS). **A**) MA plot of *Bifidobacterium* group vs the no bacteria control, DEGs of B cell activation and signaling pathways labeled. **B**) Quadrant plot showing whether DEGs have the same or opposite relationships between each of the pairwise comparisons of *Bifidobacterium* vs control and EPS vs control. Each point represents a DEG. Gene-pathway network for the top 5 over-represented GO terms in comparison of **C**) *Bifidobacterium* live vs no bacteria control and **D**) EPS vs no bacteria control. Raw counts generated by HTSeq used for differential expression (DE) analyses using DESeq2 [4] to estimate gene dispersion, normalize counts by library size and statistically determine DE genes (DEGs) using log2 fold change (LFC) >(+/-)1 and false discovery rate (FDR)<0.05. Over-representation analyses of DEGs using GO ontologies were performed using enrichGO function of clusterProfile Bioconductor package [3]. Cnetplot function used to depict the linkages of genes and GO terms as a Gene-Concept Network for over-represented GO terms based on q-value and gene-count.

**Supplemental Figure 7.** Comparison of relative abundance of bacterial groups in stool samples of the mice in different experiment groups. Cumulative relative abundance of bacteria groups from the four most abundant phyla present in all mice shown.

**Supplemental Figure 8**. Taxonomic groups differentially abundant with statistical and biological significance, ranked according to the effect size. Only taxonomic groups present at >1% in at least one sample were included in the analyses. Logarithmic linear discriminant analysis (LDA) effect size (LEfSe) was employed (Segata et al., 2011). The alpha threshold value for the pairwise non-parametric Kruskal-Wallis test was 0.05 and the threshold for the logarithmic LDA model score (Fisher, 1936) for discriminative features was 2.0. All-against-all comparison in multi-class analysis were performed.

**Supplemental Figure 9.** PCA analysis between ATCC25526 and UMB-MBP-01 groups. Canonical Correspondence Analysis (CCA) was used in ordination analysis of **A**) taxonomic profiles and **B**) functional profiles. Biplot was generated using vegan package [5, 6] based on Bray-Curtis distance. CA1 and CA2 were selected as the major components based on the eigenvalue. Microbial functional pathways were characterized using HUMAnN2 (v0.11.2)[7] and Uniref90 database [8].

**Supplemental Tables:**

**Supplemental Table 1.** Statistics of pangenome analyses. **A**) Statistics of the 79 *Bifidobacterium* *pseudolongum* strains including the 4 strains sequenced in this study; **B**) homologous gene clusters (HGCs) of *Bifidobacterium* *pseudolongum* generated in metapangenome analyses; **C**) Comparison of protein coding genes among the four bifidobacterial strains from the same source; **D**) Comparison of protein coding genes between UMB-MBP-01 and ATCC25526; **E**) HGCs enriched in different clades; **F**) Comparison of GHs (glycoside hydrolyses) between UMB-MBP-01 and ATCC25526.

**Supplemental Table 2.** Information of the genomic and metagenomes sequencing. **A**) Statistics of genomic sequencing reads and assembly; **B**) Statistics of the metagenomes sequencing of intraluminal stools.

**Supplemental Table 3.** Protein localization of the coding genes based on the presence of a signal peptide [9]. **A**) localization information of all homologous gene clusters (HGCs) of *Bifidobacterium* *pseudolongum*; **B**) Distribution of all *B. pseudolongum* HGCs localization categories; **C**) proteins predicted to be associated with cell wall; **D**) proteins predicted to be extracellular.

**Supplemental Table 4.** Information on glycosyl hydrolases (GH) characterization of *B. pseudolongum* pangenome using Carbohydrate-Active enZYmes Database (CAZy) database [10]. **A**) GHs of the HGCs generated in pangenome analyses; **B**) GH similarity among *B. pseudolongum* genomes. UMB-MBP-01 was used as the reference to compare to glycohydrolyses capabilities of all other 61 *B. pseudolongum* strains. BLAST Score Ratio (BSR) approach [1] in default setting used to calculate similarity score.

**Supplemental Table 5.** Anaerobic microplates pre-coated with various carbon sources

the carbohydrate utilization capabilities of UMB-MBP-01 and ATCC25526.

**Supplemental Table 6.** Differential expressed genes (DEGs) between each of the pairwise comparison of UMB-MBP-001 vs control and UMB-MBP-001 vs ATCC25526. **A**) List of DEGs according to bin name listed in the table correspond to the quadrant plot in **Figure 5A**; **B**) DEGs between the comparison of UMB-MBP-001 vs control; **C**) DEGs between the comparison of ATCC25526 vs control; **D**) DEGs between the comparison of UMB-MBP-001 vs ATCC25526.

**Supplemental Table 7.** Taxonomic profiles of the metagenomes sequencing of intraluminal stools. Taxonomy was characterized using the comprehensive mouse gut metagenome catalog (CMGM) [11] designed specifically to characterize mouse gut microbiome. Relative abundance of each identified bacterial groups shown in the table.

**Supplemental Table 8.** Statistics of Canonical Correspondence Analysis (CCA). CA1 and CA2 are selected as the major components based on the eigenvalue. CCA was generated using vegan package [5, 6] based on bray-curtis distance.

**Supplemental Table 9.** Primary and secondary antibodies used in this study.

**Supplemental Figure 1.**

**
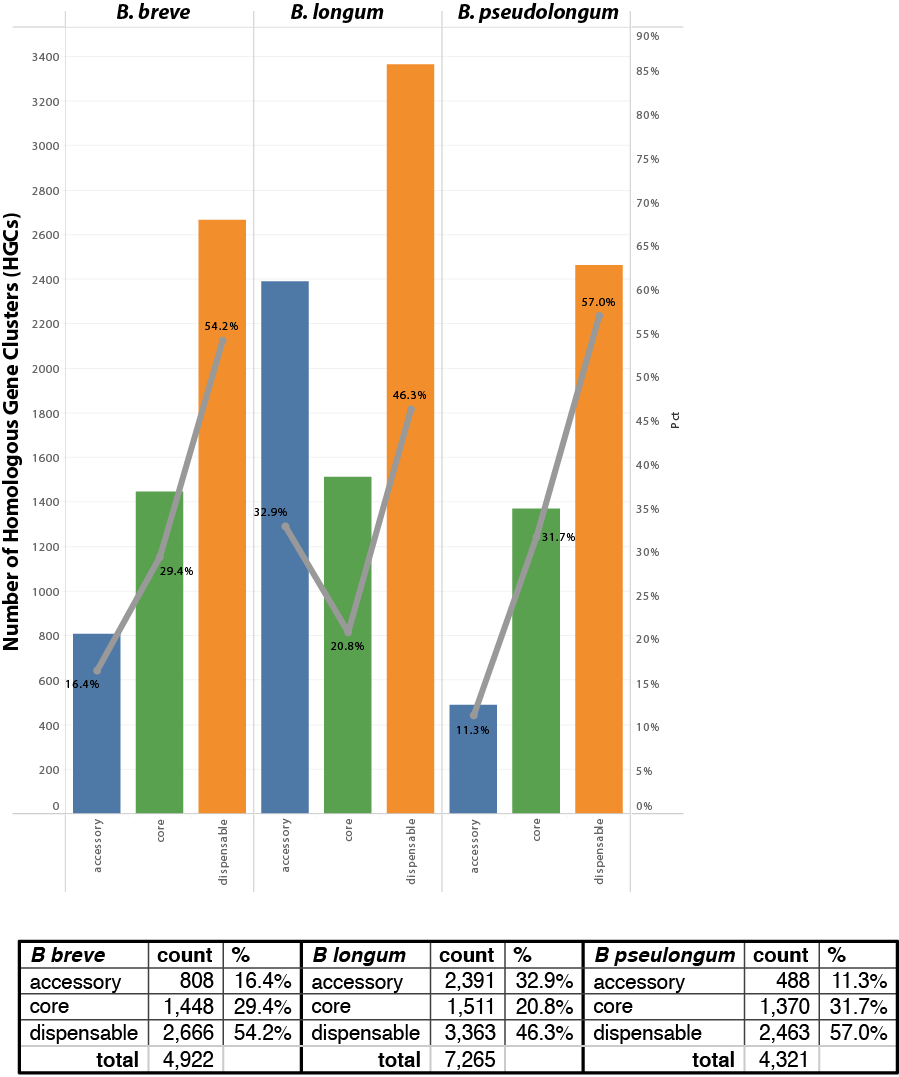
**

**Supplemental Figure 2.**

**
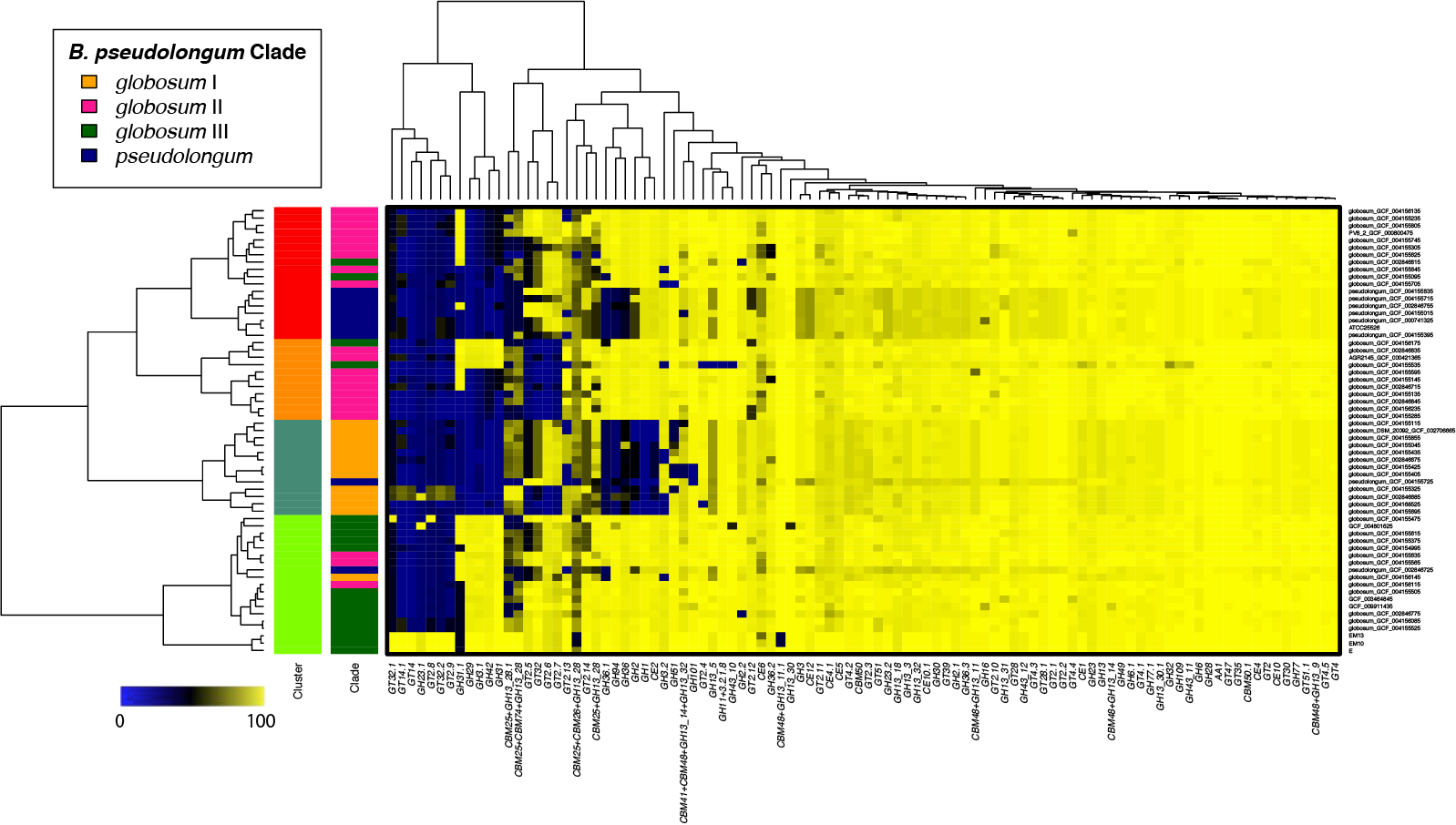
**

**Supplemental Figure 3.**

**
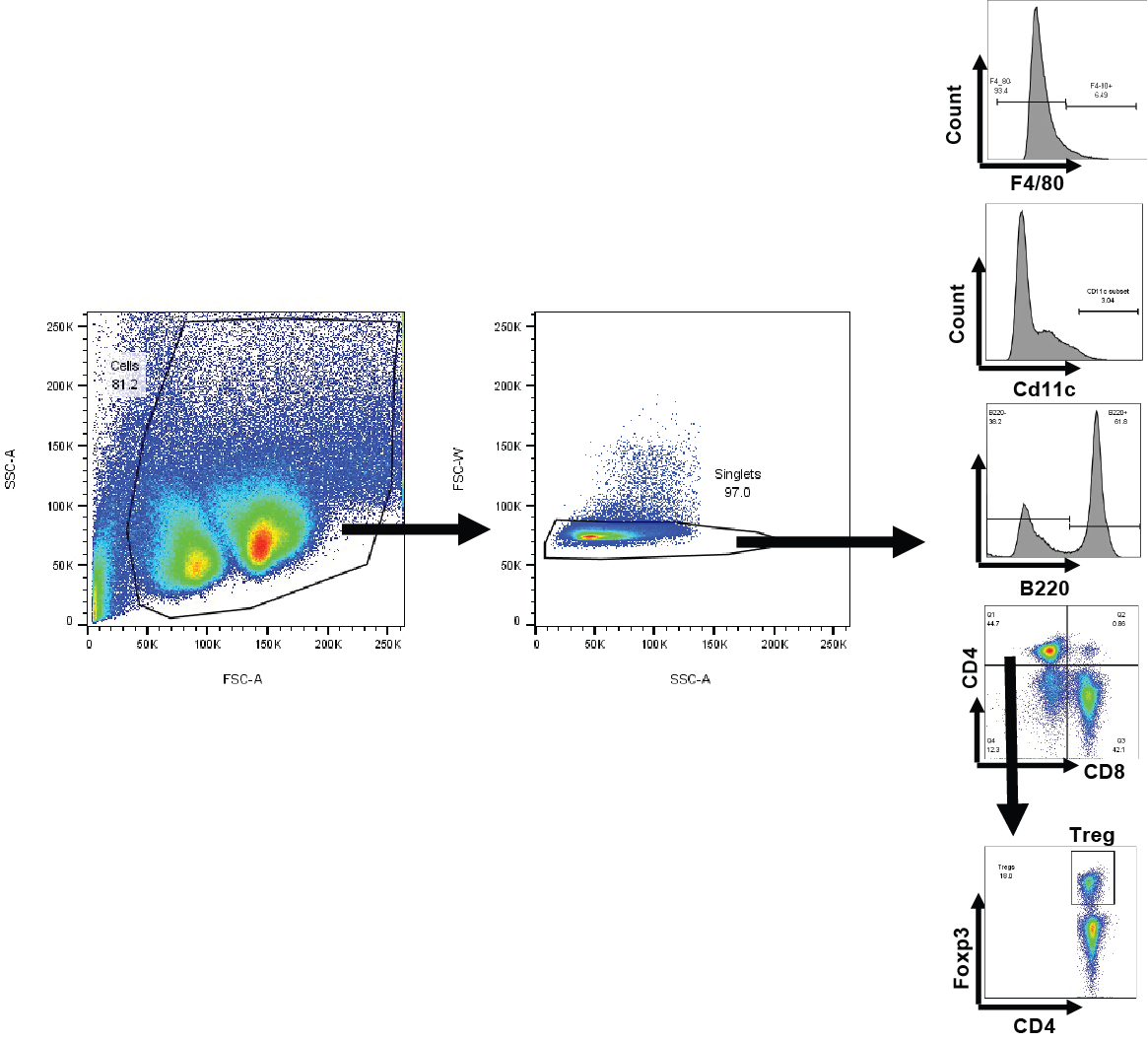
**

**Supplemental Figure 4.**


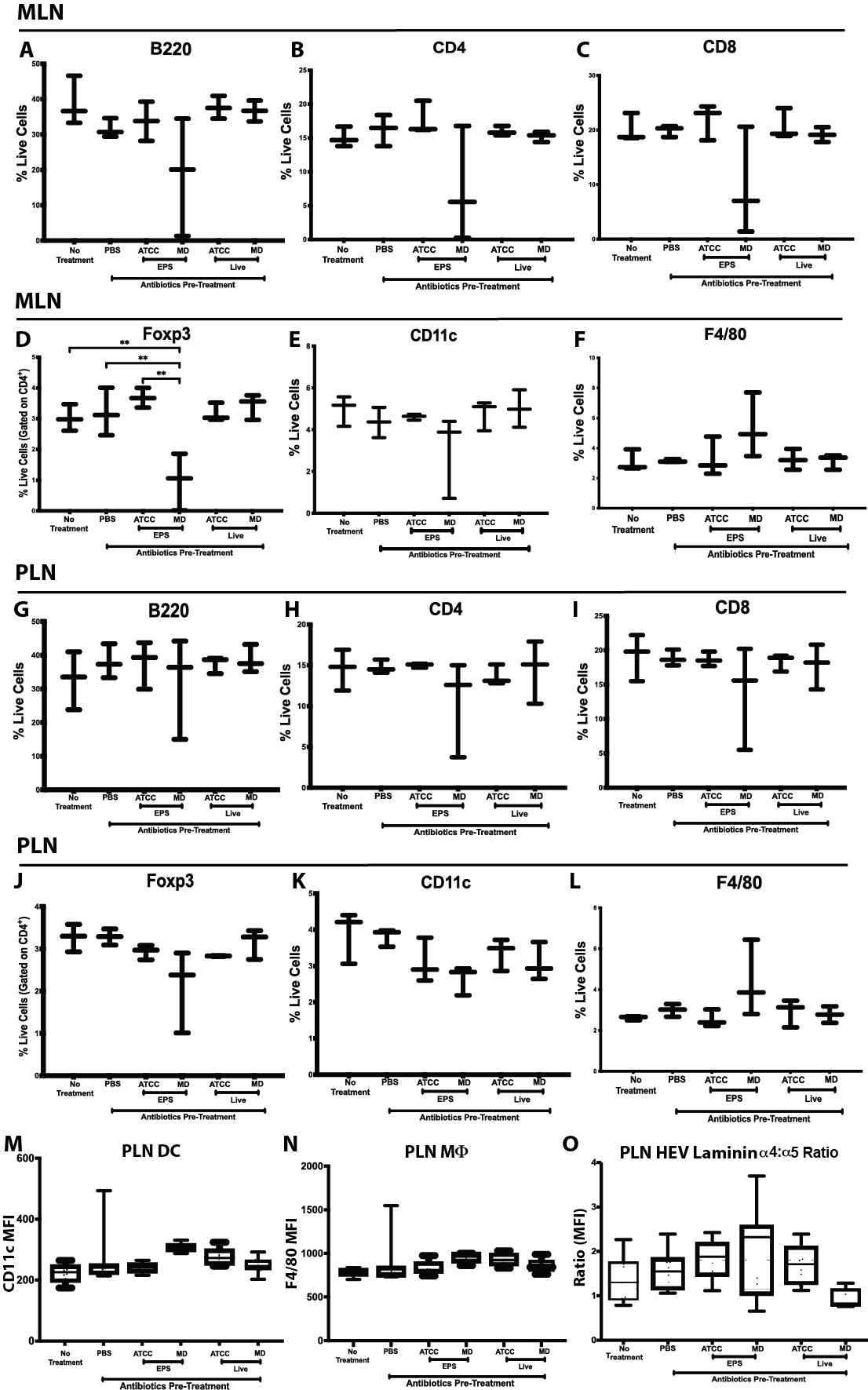
**Supplemental Figure 5.**

(Uploaded as a separate file “SFigure5.pdf”.)

**Supplemental Figure 6.**

**
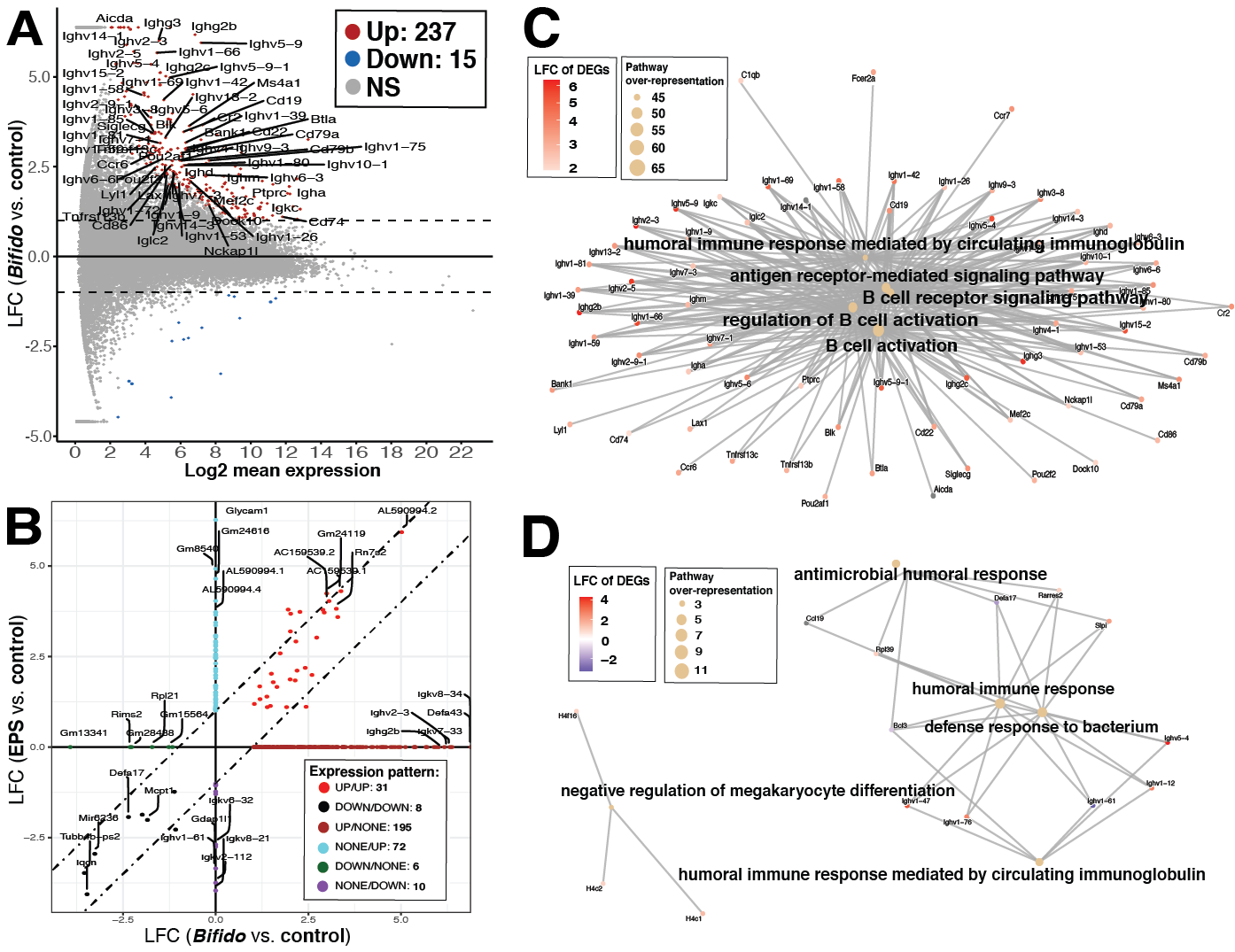
**

**Supplemental Figure 7.**


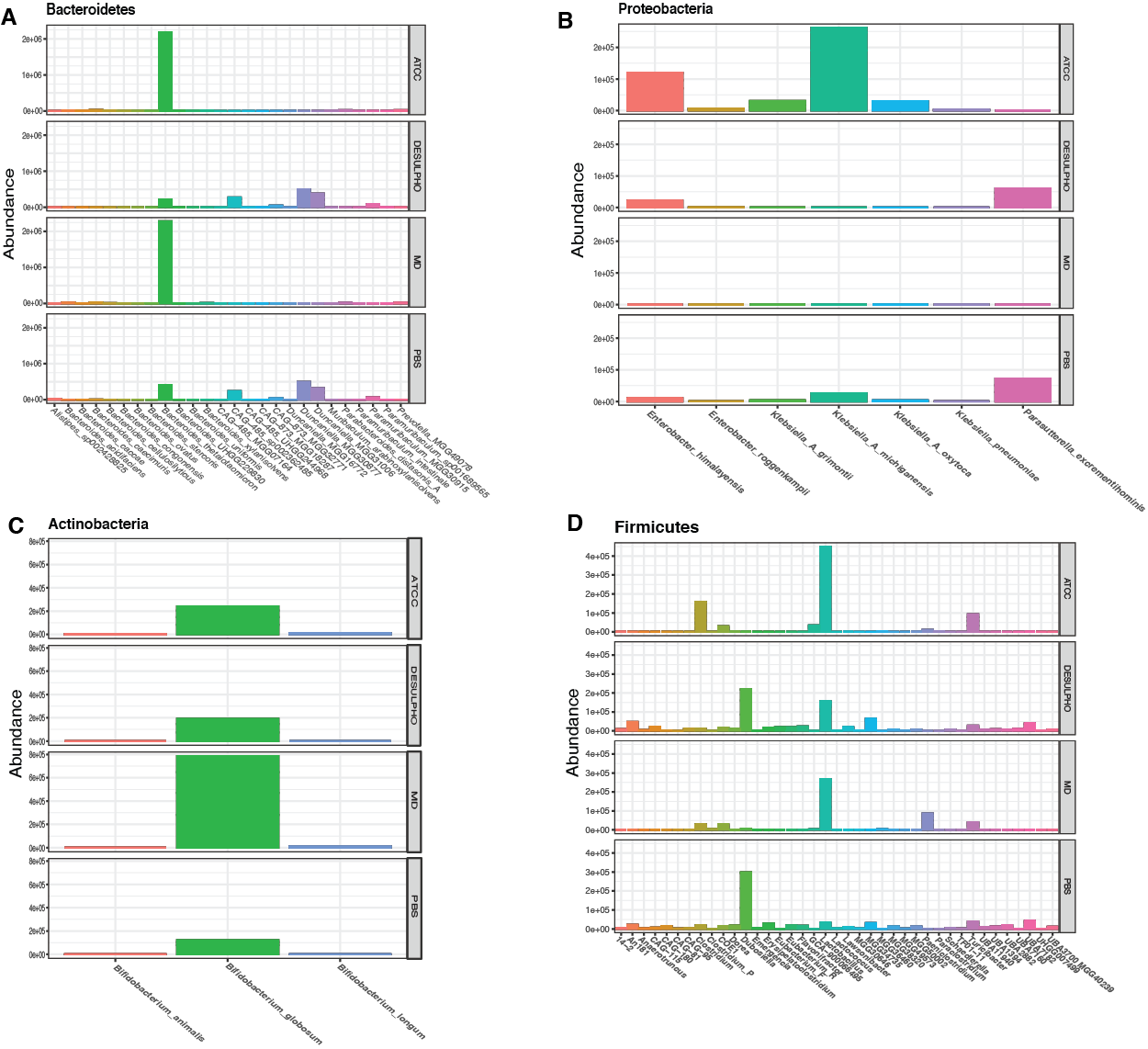


**Supplemental Figure 8.**


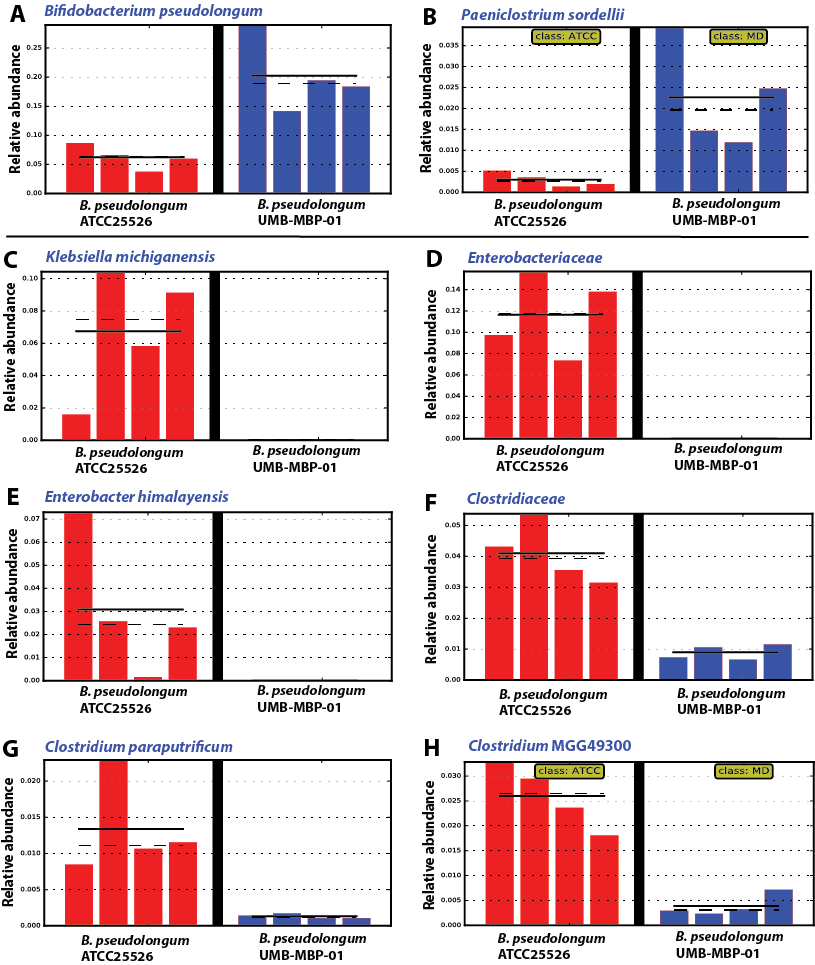


**Supplemental Figure 9.**


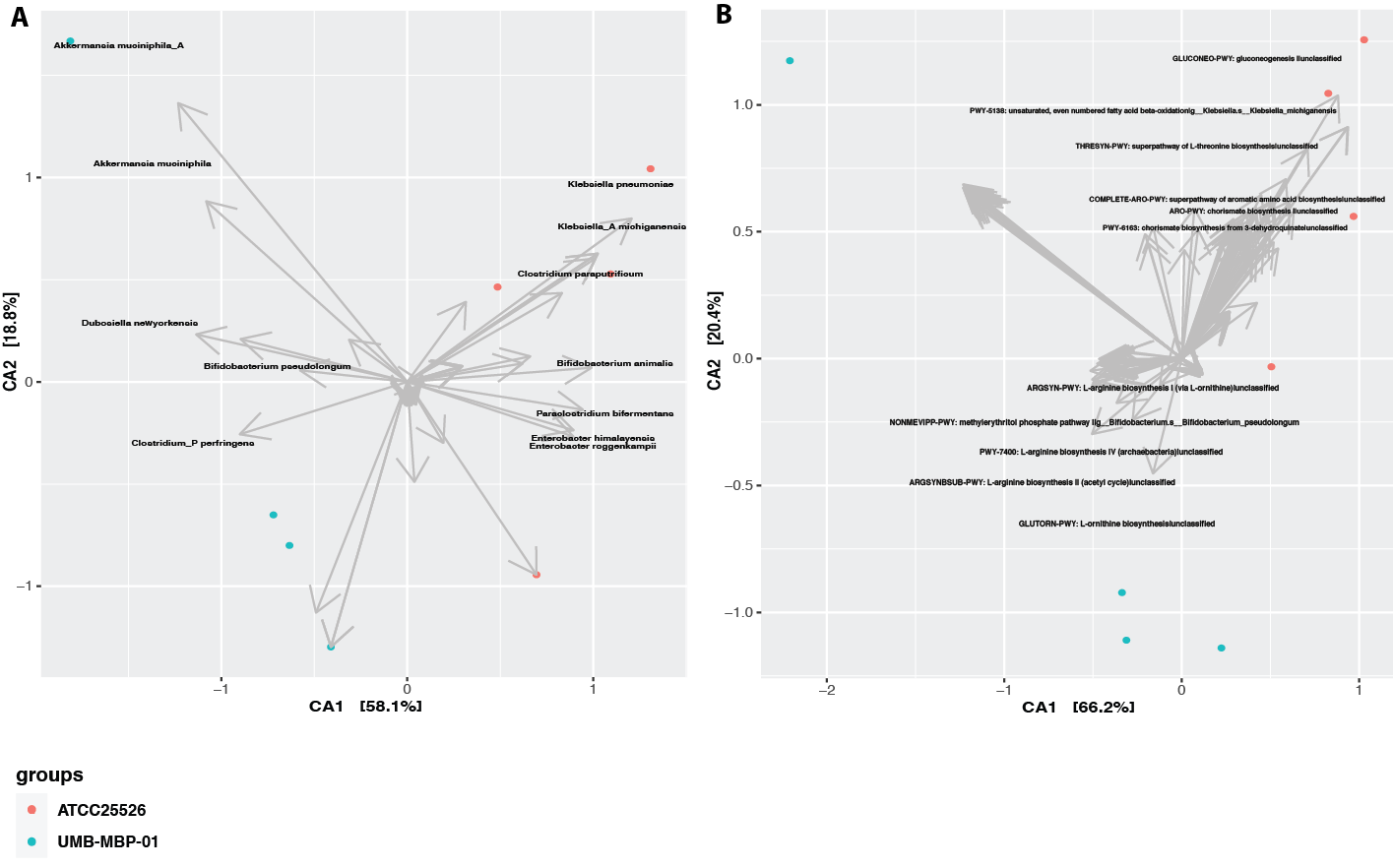


## References

1. Rasko DA, Myers GS, Ravel J: **Visualization of comparative genomic analyses by BLAST score ratio.** *BMC Bioinformatics* 2005, **6:**2.

2. Boyle EI, Weng S, Gollub J, Botstein D, Cherry JM, Sherlock G: **GO::TermFinder—open source software for accessing Gene Ontology information and finding significantly enriched Gene Ontology terms associated with a list of genes.** *Bioinformatics* 2014, **20:**3710–3715.

3. G Y, L W, Y H, Q H: **clusterProfiler: an R package for comparing biological themes among gene clusters.** *OMICS: A Journal of Integrative Biology* 2012, **16:**284-287.

4. Anders S, Huber W: **Differential expression analysis for sequence count data.** *Genome Biol* 2010, **11:**R106.

5. Jari Oksanen FGB, Michael Friendly, Roeland Kindt, Pierre Legendre, Dan McGlinn, Peter R. Minchin, R. B. O'Hara, Gavin L. Simpson, Peter Solymos, M. Henry H. Stevens, Eduard Szoecs and Helene Wagner **vegan: Community Ecology Package. R package.** version 2.4-1. edition; 2016.

6. Dixon P: **VEGAN, a package of R functions for community ecology.** *Journal of Vegetation Science* 2003, **14:**927-930.

7. Franzosa EA, McIver LJ, Rahnavard G, Thompson LR, Schirmer M, Weingart G, Lipson KS, Knight R, Caporaso JG, Segata N, Huttenhower C: **Species-level functional profiling of metagenomes and metatranscriptomes.** *Nat Methods* 2018, **15:**962-968.

8. Kanehisa M, Goto S, Sato Y, Furumichi M, Tanabe M: **KEGG for integration and interpretation of large-scale molecular data sets.** *Nucleic acids research* 2012, **40:**D109-114.

9. van Wely KH, Swaving J, Freudl R, Driessen AJ: **Translocation of proteins across the cell envelope of Gram-positive bacteria.** *FEMS Microbiol Rev* 2001, **25:**437-454.

10. Lombard V, Golaconda Ramulu H, Drula E, Coutinho PM, Henrissat B: **The carbohydrate-active enzymes database (CAZy) in 2013.** *Nucleic Acids Res* 2014, **42:**D490-495.

11. Kieser S, Zdobnov EM, Trajkovski M: **Comprehensive mouse gut metagenome catalog reveals major difference to the human counterpart.** *bioRxiv* 2021**:**2021.2003.2018.435958.
